# Quercetin inhibits the epithelial-mesenchymal transition and reverses CDK4/6 inhibitor resistance in breast cancer by regulating circHIAT1/miR-19a-3p/CADM2 axis

**DOI:** 10.1371/journal.pone.0305612

**Published:** 2024-07-11

**Authors:** Xiaogang Li, Chao Niu, Guoqiang Yi, Yuan Zhang, Wendi Jin, Zhiping Zhang, Wanfu Zhang, Bo Li

**Affiliations:** Department of General Surgery, Affiliated Hospital of Yunnan University, Kunming, Yunnan, China; University of Wisconsin-Madison, UNITED STATES

## Abstract

Breast cancer (BC) cells have a high risk of metastasis due to epithelial-mesenchymal transition (EMT). Palbociclib (CDK4/6 inhibitor) is an approved drug for BC treatment. However, the drug resistance and metastasis can impair the treatment outcome of Palbociclib. Understanding the mechanisms of EMT and Palbociclib drug resistance in BC is conducive to the formulation of novel therapeutic strategy. Here, we investigated the role of circHIAT1/miR-19a-3p/CADM2 axis in modulating EMT and Palbociclib resistance in BC. circHIAT1 and CADM2 were down-regulated in BC tissues and cell lines, and miR-19a-3p showed an up-regulation. circHIAT1 could interact with miR-19a-3p and suppress its activity, while miR-19a-3p functioned to negatively regulate CADM2. Forced over-expression of circHIAT1 could impaired the EMT status and migratory ability of BC cells, and this effect was inhibited by miR-19a-3p mimic. In addition, we also generated Palbociclib resistant BC cells, and showed that circHIAT1 and CADM2 were down-regulated in the resistant BC cells while miR-19a-3p showed an up-regulation. Forced circHIAT1 over-expression re-sensitized BC cells to Palbociclib treatment. Quercetin, a bioactive flavonoid, could suppressed the migration and invasion of BC cells, and re-sensitized BC cells to Palbociclib. The anti-cancer effect of quercetin could be attributed to its regulatory effect on circHIAT1/miR-19a-3p/CADM2 axis. *In vivo* tumorigenesis experiment further revealed that quercetin administration enhanced the anti-cancer effect of Palbociclib, an effect was dependent on the up-regulation of circHIAT1 by quercetin. In summary, this study identified quercetin as a potential anti-cancer compound to reverse Palbociclib resistance and impair EMT in BC cells by targeting circHIAT1/miR-19a-3p/CADM2 axis.

## Introduction

As the most prevalent malignancy and the top risk factor of cancer-related death in women, breast cancer (BC) is becoming a major health threat to female population [1, 2]. In spite of achievements in therapeutic approaches such as radiotherapy, surgery, endocrine therapy, chemotherapy, and targeted therapy, BC patients still suffer from metastasis and drug resistance [3, 4], which are the main causes of treatment failure and the high mortality rate. Therefore, it is imperative to uncover the factors implicated in BC cell metastasis and drug resistance development. Epithelial-mesenchymal transition (EMT) is a major biological process contributing to the metastatic features in cancer cells [5, 6]. During EMT, cancerous cells lose epithelial characteristics and gradually obtain a mesenchymal phenotype, with decreased inter-cellular adhesion and augmented migratory and invasive capacity [7, 8]. EMT process is accompanied by the down-regulation of epithelial markers including occludins, E-cadherin, and claudins, and the up-regulation of N-cadherin and vimentin [9]. Thus, suppressing EMT markers can hinder the malignant progression of BC cells.

Cyclin-dependent kinase (CDK) 4/6 are important promoter of cell cycle progression and their over-activation can induce EMT and improve cell invasion via TGF-β signaling [10]. CDK4/6 inhibitors have been developed to treat different cancers through suppressing rapid cell proliferation. The CDK4/6 inhibitor Palbociclib is a clinically approved targeted therapy for BC, which could enhance the overall survival of BC patients [10]. However, a large fraction of BC patients eventually developed resistance to CDK4/6 inhibition [11]. The joint application of CDK4/6 and aromatase inhibitors has been formulated as the first-line therapy for advanced HR+ and HER2- BC [12]. CDK4/6 inhibitor Palbociclib has been demonstrated to regulate Snail expression, a key transcription factor orchestrating EMT, and lower the risk of distant metastasis in triple negative BC [13]. Although promising results have been reported using Palbociclib at initial stage, the resistance was observed in almost all cases 24 to 28 months after the first-line therapy or shortly after the second-line therapy [14]. Overcoming CDK4/6 inhibitor resistance is a key research direction for the better management of BC.

Natural products contains a variety of structurally diversified compounds with bioactive effects. Several natural products have been used as an additive to boost chemotherapy outcome or as independent agents to lower the side effects of standard treatment [15]. Among the most promising natural products, diet-derived flavonoids have received increasing attentions as many flavonoids have been demonstrated with anti-cancer effects [16, 17]. In addition, there are several advantages in using flavonoids as adjuvant since the dietary compounds generally have low toxicity and show anti-inflammatory effects. Quercetin is identified as an active antioxidant flavonoid that can lower the level of low-density lipoproteins and attenuate the level of free radicals [18, 19]. It is widely distributed in red grapes, onion, tomato, lettuce, tea, olive oil, bracken fern, coffee, and citrus fruits. Recent evidence also showed the anti-cancer effect of quercetin in BC cells by inducing apoptotic cell death [20, 21]. Quercetin can also impair epidermal growth factor (EGF)-induced EMT and hinder the invasiveness of prostate cancer cells through PI3K/Akt signaling pathway [22]. However, whether quercetin could modulate the cellular sensitivity towards CDK4/6 inhibitor Palbociclib is unknown.

Emerging evidence has demonstrated that non-coding RNAs, such as circular RNA (circRNAs) and micro RNA (miRNAs), are implicated in regulating the malignancy of cancer cells, including the EMT and drug resistance. For example, miR-19a-3p acts as an oncogenic factor to enhance EMT and migration in BC cells [23]. Inhibiting miR-19a-3p could suppress the proliferation, EMT and migration of renal cancer cells [24]. It has also been reported that circHIAT1 may function upstream of miR-19a-3p to regulate cell growth, migration, invasion and drug sensitivity [25]. In this study, we investigated the role of circHIAT1/miR-19a-3p/CADM2 axis in modulating EMT and Palbociclib resistance in BC cells. We demonstrated the molecular interplay among circHIAT1, miR-19a-3p and Cell Adhesion Molecule 2 (CADM2) in BC cells. We dissected the roles of circHIAT1/miR-19a-3p/CADM2 axis in modulating the EMT and drug resistance of Palbociclib in BC cells. These data provide novel insights into Palbociclib drug resistance in BC and suggest quercetin as a potential bioactive compound to reverse Palbociclib resistance in BC treatment.

## Materials and methods

### Clinical sample collection

This study collected breast cancer tissues and para-cancerous specimens from breast cancer patients (N = 20 in each category) in Yunnan University Affiliated Hospital from June 2021 to April 2022. The acquisition of all clinical materials had been approved by the Ethics Committee of Yunnan University Affiliated Hospital (Approval Number: 2020092). In addition, the written form of informed consent were obtained from all the recruited subjects. All the sample handling and data processing steps were following the Declaration of Helsinki. The samples were kept in liquid nitrogen and then at -80°C for later experiments.

### Cell culture

The normal human breast epithelial cell line (MCF-10A) and human BC cell lines (MDA-MB-231 and MCF-7) were procured from Hongshun Biotechnology (Shanghai, China), authenticated using STR profiling, and tested to be mycoplasma-free by the supplier. DMEM medium was utilized to culture the cells, with streptomycin (100 μg/ml), penicillin (100 U/ml), and 10% FBS (Yeasen, Beijing, China). The cells were maintained at 37°C with 5% CO_2_ in a humidified chamber.

### Resistant cell line generation

BC cell lines (MDA-MB-231 and MCF-7) were treated with increasing doses of palbociclib (0.1, 0.2, 0.4, 0.8, 1 μM, MedChemExpress, Shanghai, China), with each concentration for 2 to 3 weeks. The drug-containing medium was replenished every three days. Cells were sub-cultured every week when the cell density reached about 80%. After six months, drug-resistant cells were then maintained in the complete medium with 0.8 μM palbociclib. Quercetin was purchased from MedChemExpress (Shanghai, China).

### Cell transfection

Lipofectamine^™^2000 (Invitrogen, Shanghai, China) transfection reagents were used to transfect expression plasmid, siRNA, miRNA mimic or inhibitor into BC cells. Control siRNA and siRNA targeting circHIAT1 were purchased from Genepharma (Shanghai, China). miRNA mimic, inhibitor or the corresponding controls were synthesized by RiboBio Biotech (Guangzhou, China). The empty vector (pcDNA3.1) and circHIAT1 expression vector (pcDNA3.1-circHIAT1) were produced by Sangon Biotech (Shanghai, China). Briefly, cells were seeded in 6-well plates at a density of 5x10^5 cells/well. 24 h later, 100 nm of siRNA, miRNA mimic or 6 μg of plasmid was added into 100 μl Opti-MEM^®^ I Reduced-Serum Medium (Invitrogen, Shanghai, China), and then 6 μL Lipofectamine 2000 reagent was added in to the medium for 10 min incubation at ambient temperature. The mixture was added to cell culture for 48 transfection before further experimental analysis.

### qRT-PCR

Following the manufacturer’s instructions, total RNA was extracted using TRIzol (Invitrogen, CA, USA) for quantitative real-time polymerase chain reaction (qRT-PCR). The supernatant was mixed with an equal volume of isopropyl alcohol for 10 minutes before being centrifuged at 12000g/min at 4°C for 10 minutes. After that the upper layer of clear solution was discarded, the precipitation was washed with 75% ethanol (7500g/min, 4°C, 5min). The remaining RNA samples were dissolved using DEPC water and the concentration was measured using a spectrophotometer. One Step PrimeScript miRNA cDNA Synthesis Kit (Roche, Laussane, Switzerland) was used to for cDNA synthesis from 1 μg of total RNA. AceQ^®^ qPCR SYBR^®^ Green Master Mix (Vazyme, Beijing, China) was used for qPCR detection on the 7500 Real Time PCR System (Applied Biosystems, CA, USA). Actb or U6 gene was used as the internal reference for target gene normalization by 2^-ΔΔCt^ method. All primers were synthesized by Anhui Universal Biosynthesis (Anhui, China).

### Western blot

Using a protein extraction kit (Yeasen, Beijing, China), total protein was extracted fro cultured cells and the protein concentration was determined using a BCA assay kit (Beyotime, Beijing, China). 10 μg of protein was analyzed using 12% polyacrylamide gels, and then deposited to polyvinylidene fluoride (PVDF) membranes (Sangon Biotech, Shanghai, China). PVDF membranes was blocked with 5% nonfat milk at room temperature for 1 h. The PVDF membrane was then treated overnight at 4°C with the primary antibodies against the target proteins, followed by a 1-hour incubation with the detection secondary antibody. A Beyo ECL Star kit was employed to detect the protein bands (Beyotime, Beijing, China). The following antibodies were used in this study: N-cadherin, E-cadherin, CADM2, Actin, GAPDH (at 1:1000 dilutions, Cell Signaling Technology, CA, USA). Anti-rabbit IgG HRP (1:5,000; Abcam, Cambridge, UK) was used as the secondary antibody. Protein bands were imaged using a gel imager system (Bio-Rad, CA, United States).

### Immunohistochemistry (IHC)

The collected tumor tissues were prepared into 5 μm sections in paraffin. After deparaffinization and rehydration, antigen retrieval was performed by heating the section in citrate unmasking solution (SignalStain^®^ Citrate Unmasking Solution (10X) (#14746), Cell Signaling Technologies, CA, USA) at 95°C for 10 minutes. Sections were cooled on bench top for 30 min, and the washed in dH2O three times for 5 minutes each. The sections were further incubated in 3% hydrogen peroxide for 10 minutes, and then blocked for 1 hour at room temperature in TBST buffer with 5% normal Goat Serum. The following primary antibodies were used for staining: anti-CADM2 and anti-Ki-67 (1:200, Abcam, Cambridge, UK) for 18 hour at 4°C. The section was then soaked with 500 μL of HRP anti-rabbit secondary antibody (1: 2000 dilution, Cell Signaling Technologies) for 30 minutes at ambient temperature, followed by the addition of 500 μl SignalStain^®^ substrate (#8059, Cell Signaling Technologies) for 5 minutes. After rinsing with dH_2_O two times, the section was counter-stained by hematoxylin for 1 minute, and the images were recorded using a light microscope (CX23, Olympus, Japan).

### Luciferase reporter assay

The wild type binding sites between circHIAT1/miR-19a-3p and miR-19a-3p/CADM2 mRNA 3’UTR were predicted by Starbase (https://starbase.sysu.edu.cn/starbase2/) or Tarrget Sacn online tools (http://www.targetscan.org/TargetScan). The corresponding wild type (WT) binding sites or mutated binding sites (MUT) were cloned into the pmirGLO luciferase reporter vector (E1330, Promega, WI, USA). The WT or MUT luciferase reporter was co-transfected into MCF-7 cells together with miR-NC or miR-19a-3p mimic using Lipofectamine 2000. After 48 hours, 100 uL of 1xPLB passive buffer (Luc-Pair Duo-Luciferase Assay Kit, GeneCopoeia, Najing, China) was added to each well of 96-well plates, and the solution was slowly agitated at ambient temperature for 10 minutes. The lysate was centrifuged for 15 minutes at 12000 rpm/min at 4°C. 100 uL assay working solution was mixed with an equal volume of lysate for 3 times. After 15 minutes, the Firefly Luciferase and Renilla Luciferase activity was developed using ABP dual luciferase assay kit (Chemstan, Wuhan, China).

### Biotinylated RNA pull-down analysis

Lipofectamine^™^2000 Reagent was employed to introduce biotinylated miR-NC probe (50 nM) or miRNA mimic into MCF-7 cells. 48 h post transfection, the cell lysate from 5x10^5^ MCF-7 cells was collected and subjected to the incubation with 100 uL C-1 streptavidin magnetic beads (65001, Life Technologies, Shanghai, China) for 4 h with rotation at 4°C. After washing, the associated RNA molecules on the beads was extracted using TRIzol reagent, and the relative enrichment of circHIAT1 and CADM2 mRNA was quantified through qRT-PCR.

### Cell counting kit-8 (CCK-8) assay

Cells in different experiment groups were inoculated in 96 culture plate (1500 cells per well) and maintained in the incubator for different duration. 10 uL CCK-8 reagent (Yeasen, Beijing, China) was then loaded to each well and 3-hour incubation (37°C, 5% CO_2_) after being incubated for indicated duration. The culture medium was then discarded and the resulted formazan crystal was dissolved in 150 uL DMSO for 15 minutes. A microplate detector was utilized to record the data at 450nm (OD).

### Scratch assay

Cells were grown in a 12-well plate to reach 90% confluency. A sterile pipette tip was utilized to establish a the middle area of cell monolayer. Floating cells were washed away with medium and the remain cells were cultured for another 18 h. The wound closure situation was observed under a light microscope.

### Transwell invasion assay

Matrigel matrix (Corning, CA, USA) was applied in a 12-well Transwell chamber and air-dried at 37°C. 500 uL serum-free medium containing 2.5x10^5^ cells were added into the upper chamber, with 500 uL complete medium being loaded to the lower well. Cells were incubated for 24 hours, and the invading cells on the membrane were fixed with cold 75% ethanol and then labeled with 0.1% crystal violet (Yeasen, Beijing, China) for 30 minutes. At the end of staining, 33% acetic acid was used for decolorization and the images were recorded under a microscope.

### Lactate dehydrogenase (LDH) release assay

LDH activity was detected with Lactate Dehydrogenase Assay kit (Beyotime, Beijing, China). Briefly, LDH assay buffer and LDH substrate reaction were mixed and added into each standard of LDH, supernatant from each sample and positive control well. Standard wells = 50 μL standard dilutions; Sample wells = 50 μL samples (adjust volume to 50 μL/well with LDH Assay Buffer); Positive control = 5 μL Positive control and adjust volume to 50 μL/well with LDH Assay Buffer. 50 μL of Reaction Mix was added into each standard, sample and positive control sample well. After gentle mix, the output was measured immediately at OD 450 nm using a microplate reader at 37°C.

### Xenograft mouse model

The Department of Experimental Animal model at Yunnan University provided pathogen-free environments for raising BALB/C nude female mice (5 to 6-week-old). The nude mice weighing 30–40 g were housed in pathogen-free conditions on a 12-h light/dark cycle with free access to food and water. The mice were randomly assigned to 5 groups (5 mice in each group): control MCF-7 injection; resistant MCF-7 injection; resistant MCF-7 injection+ quercetin (50 mg/kg); resistant MCF-7 injection+quercetin and control siRNA; resistant MCF-7 injection+quercetin+circHIAT1 siRNA. 2×10^6^ MCF-7 cells in 0.15 ml PBS was administered subcutaneously at the right flank of each mouse. 0.5 mg 17β-estradiol (E2) pellet was implanted into each mouse to support the tumor formation of MCF-7 cells. The administration of quercetin (50 mg/kg/week) and siRNA (500 μg/kg/week) was through intraperitoneal injection. On day 28 following cell injection, all the mice were euthanized by CO_2_ asphyxiation and the death was assured by subsequent cervical dislocation. The tumors of terminally dead mice were resected for weight measurement and further analysis. The experimental protocols had been approved by Yunnan University Animal Center Animal Welfare Committee (Approval Number: YNU20200296).

### Statistical analysis

Data analyses were performed in SPSS (v23.0) and GraphPad Prism (v6.0). The findings were reported using mean±SD from at least 3 independent experiments. The statistical difference between two groups was compared using unpaired student’s t tests. Comparisons among multiple groups were analyzed using one-way analysis of variance (ANOVA) with Tukey’s post hoc test for pairwise comparison. Comparisons of data at multiple time points were examined using two-way ANOVA. P < 0.05 was considered to be statistically different.

## Results

### circHIAT1 negatively targets miR-19a-3p in BC

**Fig 1:**
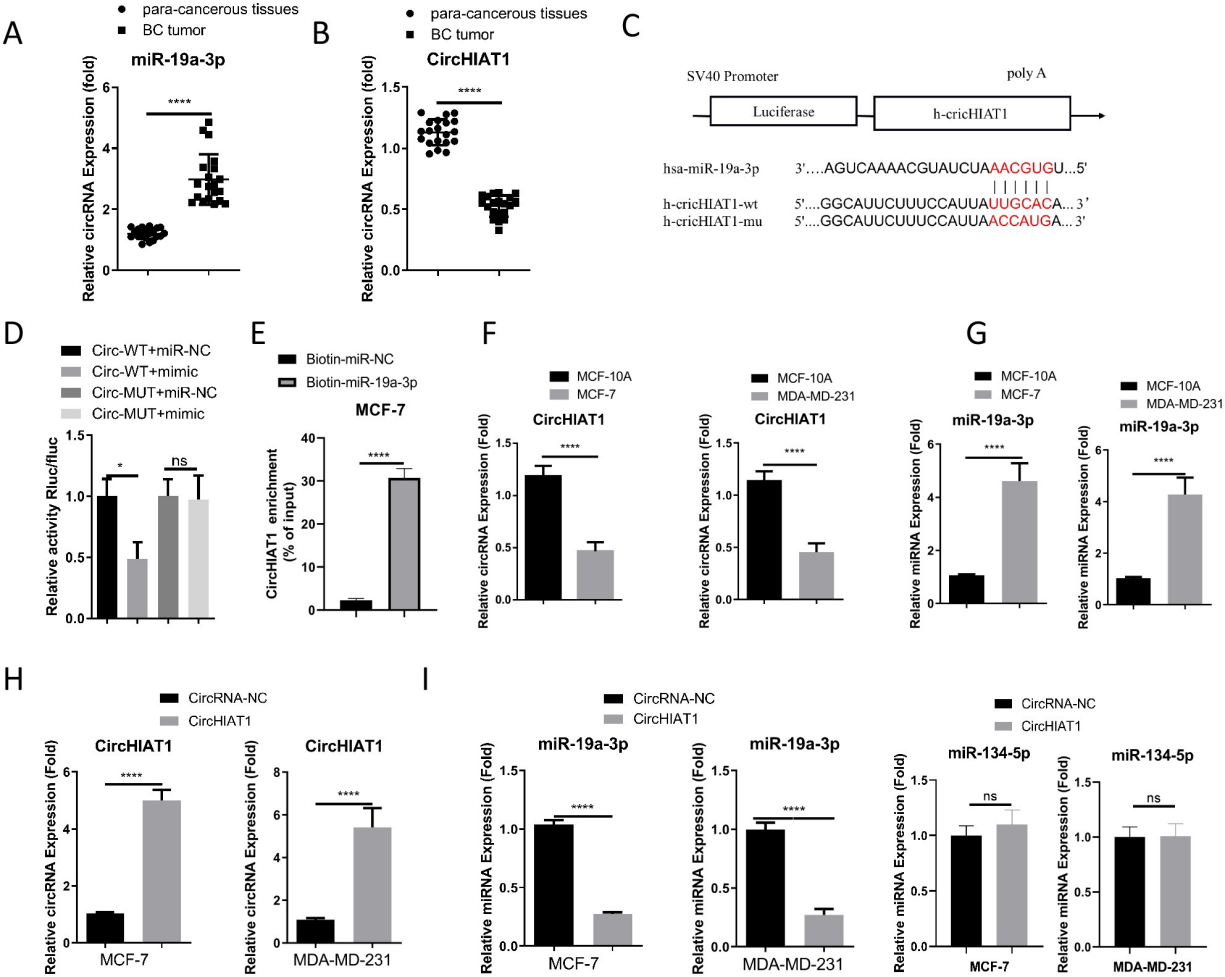
circHIAT1 negatively targets miR-19a-3p in BC. (A). qRT-PCR analysis of circHIAT1 levels in BC tumor and para-cancerous normal tissues (n = 20 pairs); (B). qRT-PCR analysis of miR-19a-3p levels in BC tumor and para-cancerous normal tissues (n = 20 pairs); (C). Starbase prediction of interacting sites between circHIAT1 and miR-19a-3p; (D). Dual luciferase reporter assay using circHIAT1 WT or MUT reporter in MCF-7 cells; (E) RNA pull-down analysis using biotin-labeled miR-NC (negative control) or miR-19a-3p probe in MCF-7 cells. qRT-PCR was conducted to examine the relative enrichment of circHIAT1. (F). qRT-PCR analysis of circHIAT1 levels in MCF-10A (normal breast epithelial cell) and BC cell lines (MCF-7 and MDA-MB-231); (G). qRT-PCR analysis of miR-19a-3p levels in MCF-10A (normal breast epithelial cells) and BC cells (MCF-7 and MDA-MB-231); (H) qRT-PCR analysis of circHIAT1 levels after the transfection of empty vector or circHIAT1 expression vector; (I). qRT-PCR analysis of miR-19a-3p and an unrelated miRNA (miR-134-5p, not predicted to interact with circHIAT1) expression levels after the transfection of empty vector or circHIAT1 expression vector. *p<0.05; **p<0.01; ***p<0.001; ****p<0.0001.

We first examined the relative expression of circHIAT1 and miR-19a-3p in tumor specimens and the para-cancerous tissues collected from breast cancer patients in our study (N = 20 in each category). qRT-PCR analysis showed that circHIAT1 was down-regulated, while miR-19a-3p showed an up-regulation in BC tumor samples (Fig 1A and 1B). Through Starbase prediction, there were potential interaction sites between circHIAT1 and miR-19a-3p (Fig 1C). We then performed dual luciferase assay using the reporter vector wild type (WT) or mutated binding sites (MUT). The over-expression of miR-19a-3p using miR-19a-3p mimic could suppress the activity of WT reporter, while there was no inhibition for the MUT reporter (Fig 1D), indicating the interaction between circHIAT1 and miR-19a-3p at predicted binding sequences. To further demonstrate their physical interaction, we performed RNA pull-down assay using biotin-labeled miR-NC (negative control) or miR-19a-3p probe. We showed that compared to miR-NC probe, biotinylated miR-19a-3p precipitated a significantly higher level of circHIAT1 (Fig 1E). Together, these data suggest the physical association between miR-19a-3p and circHIAT1. Compared to normal breast cell line MCF-10A, circHIAT1 expression level was reduced in BC cell lines (MDA-MB-231 and MCF-7), while miR-19a-3p was up-regulated in BC cell lines (Fig 1F and 1G). We next transfected empty vector or circHIAT1 expression vector in BC cell lines, and the circHIAT1 vector could significantly increase circHIAT1 level (Fig 1H). Upon circHIAT1 over-expression, the level of miR-19a-3p was significantly repressed in BC cell lines, while the expression level of an unrelated miRNA (miR-134-5p, not predicted to interact with circHIAT1) was not affected (Fig 1I). Collectively, these findings indicate that circHIAT1 interacts with miR-19a-3p to negatively regulate its level in BC cells.

### circHIAT1/miR-19a-3p axis regulates CADM2 expression in BC cells

**Fig 2:**
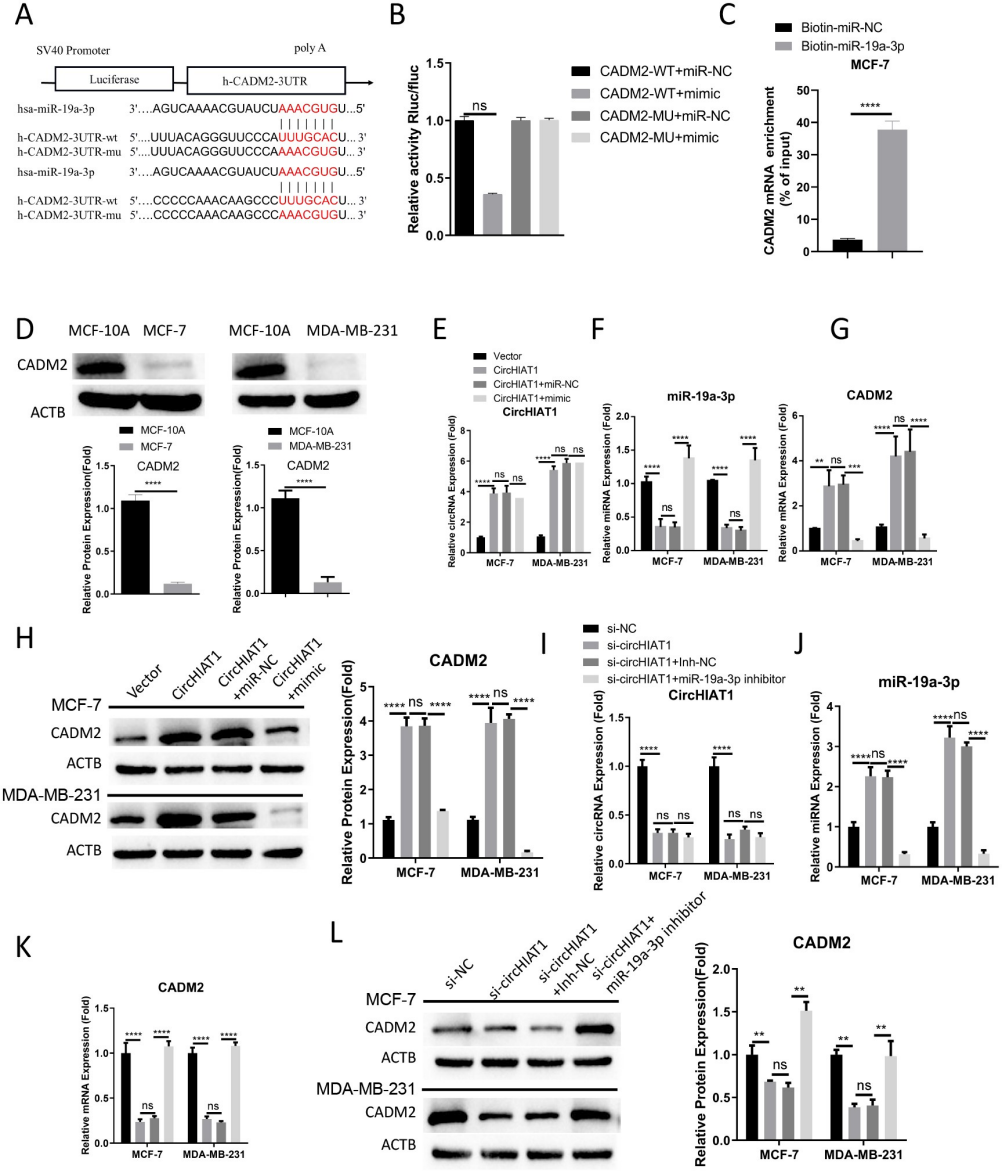
circHIAT1/miR-19a-3p axis regulates CADM2 expression in BC cells. (A). Starbase prediction of interacting sites between miR-19a-3p and CADM2 mRNA 3’UTR; (B). Dual luciferase reporter assay using CADM2 WT or MUT reporter; (C). RNA pull-down analysis using biotin-labeled miR-NC (negative control) or miR-19a-3p probe in MCF-7 cells. qRT-PCR was conducted to examine the relative enrichment of CADM2 mRNA; (D) Western blot analysis of CADM2 level in MCF-10A (normal breast epithelial cells) and BC cells (MCF-7 and MDA-MB-231). (E-H). BC cells were transfected with empty vector, circHIAT1 expression vector, circHIAT1 expression vector+miR-NC, or circHIAT1 expression vector+miR-19a-3p mimic. qRT-PCR was conducted to analyze the relative expression of (E) circHIAT1, (F) miR-19a-3p, (G) CADM2 mRNA; (H) Western blot analysis of CADM2 protein levels in above conditions; (I-L). BC cells were transfected with si-NC, si-circHIAT1, si-circHIAT1+Inh-NC, or si-circHIAT1+miR-19a-3p inhibitor. qRT-PCR was conducted to analyze the relative expression of (I) circHIAT1, (J) miR-19a-3p, (K) CADM2 mRNA; (L) Western blot analysis of CADM2 protein levels in above conditions. *p<0.05; **p<0.01; ***p<0.001; ****p<0.0001.

TargetScan tool was used to predict the potential targets of miR-19a-3p. The results indicated that there were potential binding sites for miR-19a-3p at the 3’UTR of CADM2 mRNA (Fig 2A). Dual luciferase reporter assay confirmed that miR-19a-3p interacts the 3’UTR of CADM2 mRNA at predicted wildtype sequences (Fig 2B). RNA pull-down analysis further revealed the enrichment of CADM2 mRNA by biotin-labeled miR-19a-3p probe (Fig 2C). Besides, CADM2 expression level was significantly reduced in BC cell lines in comparison to MCF-10A cells (Fig 2D). To examine the interplay of circHIAT1 and miR-19a-3p on CADM2 expression, BC cells were transfected with empty vector, circHIAT1 expression vector, circHIAT1 expression vector+miR-NC, or circHIAT1 expression vector+miR-19a-3p mimic. circHIAT1 expression vector transfection increased circHIAT1 expression, which was not affected by miR-19a-3p mimic (Fig 2E). circHIAT1 over-expression repressed miR-19a-3p level, and the co-introduction of miR-19a-3p mimic rescued miR-19a-3p expression (Fig 2F). circHIAT1 over-expression could also lead to CADM2 up-regulation at both protein and mRNA level, while the co-transfection of miR-19a-3p mimic suppressed the effect of circHIAT1 (Fig 2G and 2H). In contrast, when BC cells were transfected with si-NC, si-circHIAT1, si-circHIAT1+Inh-NC, or si-circHIAT1+miR-19a-3p inhibitor, the silencing of circHIAT1 caused the up-regulation of miR-19a-3p and the down-regulation of CADM2. These effects were reversed by miR-19a-3p inhibitor (Fig 2I–2L). Therefore, circHIAT1/miR-19a-3p axis regulates CADM2 expression in BC cells.

### circHIAT1/miR-19a-3p/CADM2 axis modulate EMT status and the mobility of BC cells

**Fig 3:**
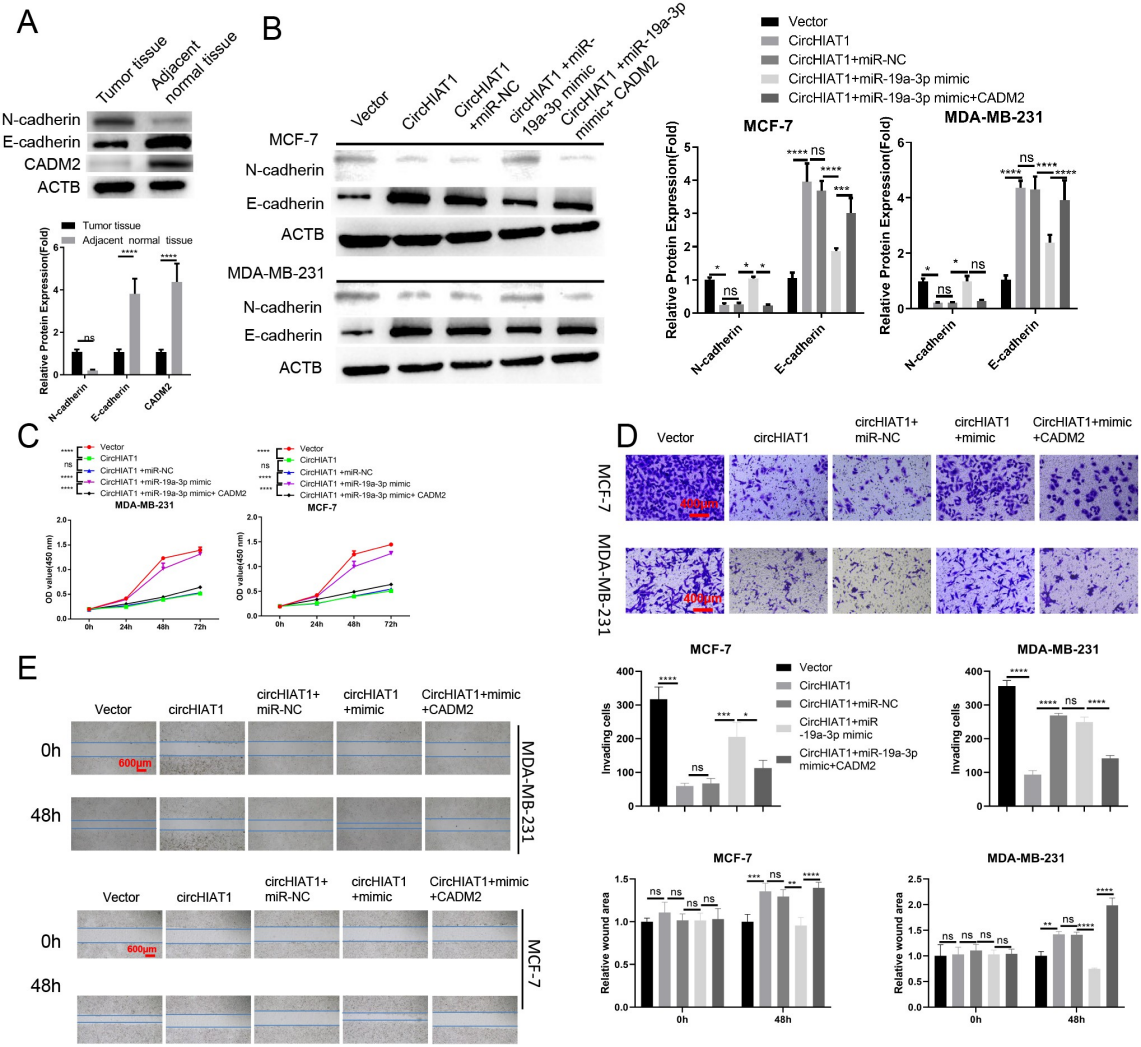
circHIAT1/miR-19a-3p/CADM2 axis modulate EMT status and the mobility of BC cells. (A). IHC staining of CADM2, E-cadherin and N-cadherin in BC tumor and para-cancerous tissues. BC cells were transfected with empty vector, circHIAT1 expression vector, circHIAT1 expression vector+miR-NC, circHIAT1 expression vector+miR-19a-3p mimic, or circHIAT1 expression vector+miR-19a-3p mimic+ CADM2 expression vector. (B). Western blot analysis of E-cadherin and N-cadherin in above conditions. (C). CCK-8 proliferation assay (D). Transwell invasion assay and (E). Scratch assay were performed in above groups of BC cells. *p<0.05; **p<0.01; ***p<0.001; ****p<0.0001.

Entranced mitigation and invasiveness contributed to metastasis in BC cells due to the EMT [5, 6]. We examined the EMT markers (E-cadherin and N-cadherin) in BC tumor tissues and the para-cancerous samples. The results revealed an increased expression of N-cadherin and a reduced level of E-cadherin in the tumor samples, indicating the enhanced mesenchymal feature in BC. Meanwhile, CADM2 expression also displayed a down-regulation in the tumor samples (Fig 3A). We next examined whether circHIAT1/miR-19a-3p axis impacts on the migratory and invasion ability through EMT in BC cells. circHIAT1 over-expression reduced N-cadherin level and promoted the expression of E-cadherin, while the co-administration of miR-19a-3p mimic abrogated the effects of circHIAT1 over-expression; however, the co-transfection of CADM2 vector reversed the effect of miR-19a-3p mimic (Fig 3B). CCK-8 assay revealed that circHIAT1 over-expression caused the retardation of cell proliferation, which could be rescued by miR-19a-3p mimic; the co-transfection of CADM2 vector repressed the proliferation (Fig 3C). Consistent with the change of EMT markers, circHIAT1 over-expression suppressed cell migration and invasion in the cell scratch and transwell invasion assays. The co-transfection of miR-19a-3p mimic largely rescued the migration and invasion abilities, while the effect of miR-19a-3p mimic was abolished by CADM2 expression (Fig 3D and 3E) These data imply that circHIAT1/miR-19a-3p axis modulates cell migration and invasion by regulating EMT in BC cells through targeting CADM2.

### Quercetin suppresses migration and invasion of BC cells at sub-toxic dose

**Fig 4:**
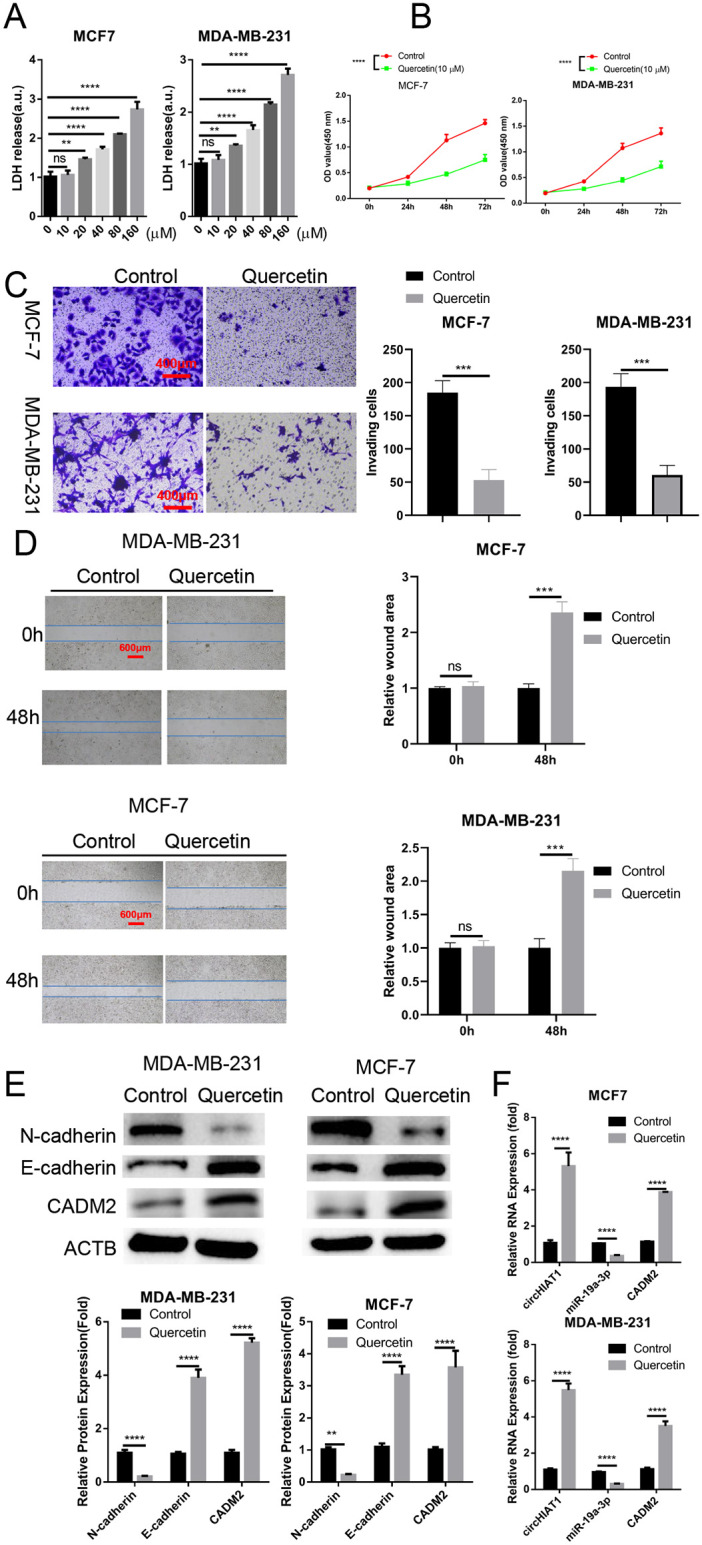
Quercetin suppresses migration and invasion of BC cells at sub-toxic dose. (A). Cytotoxicity of quercetin on MDA-MB-231 and MCF-7 cell lines were assessed by LDH release assay after treating the cells with 0 μM, 10 μM, 20 μM, 40 μM, 80 μM, 160 μM quercetin for 48 hours; (B). At 10 μM of quercetin, CCK-8 assay was performed to determine the anti-proliferation effect of quercetin in BC cells; (C). Transwell invasion assay (D). Scratch assay was performed in BC cells treated with 10 μM of quercetin for 48 hours; (E). Western blot analysis of CADM2, E-cadherin and N-cadherin in BC cells treated with 10 μM of quercetin for 48 hours; (F). qRT-PCR analysis of CADM2, circHIAT1 and miR-19a-3p in BC cells treated with 10 μM of quercetin for 48 hours. *p<0.05; **p<0.01; ***p<0.001; ****p<0.0001.

Quercetin was reported to have anti-cancer effect in BC cells [20, 21], while its potential effect on EMT has not been investigated. We first examined the cytotoxicity of quercetin on MDA-MB-231 and MCF-7 cell line. Cells were treated with 0 μM, 10 μM, 20 μM, 40 μM, 80 μM, 160 μM quercetin for 48 hours and the cell toxicity was assessed by LDH assay. The results showed that quercetin showed significant cytotoxicity above 20 μM, while at 10 μM it did not exert significant toxicity on BC cells (Fig 4A). We therefore selected 10 μM as the sub-toxic concentration in following experiments, which excluded the potential toxic effects of quercetin. At 10 μM, CCK-8 assay demonstrated that quercetin could show anti-proliferation effect on BC cells (Fig 4B). Besides, quercetin also suppressed the migration and invasion in BC cells at the sub-toxic concentration (Fig 4C and 4D). Western blot analysis of E-cadherin and N-cadherin also showed that quercetin repressed EMT process in BC cells (Fig 4E). We also analyzed the impact of quercetin on the expression of circHIAT1, miR-19a-3p and CADM2. Intriguingly, quercetin treatment promoted the expression of circHIAT1 and CADM2, but reduced miR-19a-3p level in BC cells (Fig 4E and 4F). These results prompted us to further study whether circHIAT1/miR-19a-3p axis mediates the effect of quercetin.

### The anti-cancer effect of quercetin depends on circHIAT1/miR-19a-3p axis

**Fig 5:**
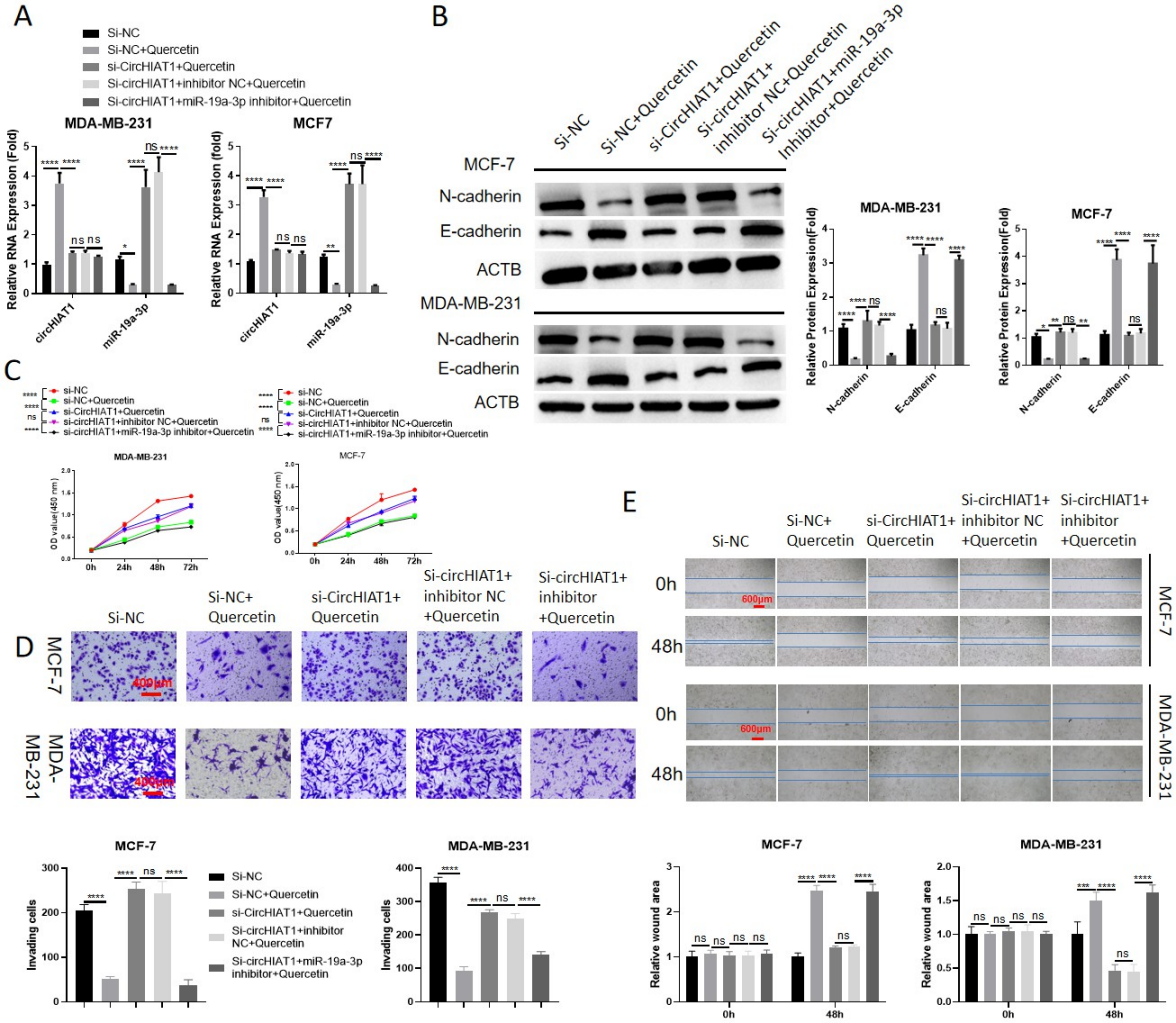
The anti-cancer effect of quercetin depends on circHIAT1/miR-19a-3p axis. MCF-7 cells were treated with 10 μM quercetin in the presence of control siRNA, circHIAT1 siRNA or circHIAT1 siRNA plus miR-19a-3p inhibitor. (A). qRT-PCR was performed to analyze circHIAT1 and miR-19a-3p expression; (B) Western blot analysis of CADM2, E-cadherin and N-cadherin; (C). CCK-8 proliferation assay; (D). Transwell invasion assay; and (E). Scratch assay in above conditions.*p<0.05; **p<0.01; ***p<0.001; ****p<0.0001.

In MCF-7 cells, we treated the samples with 10 μM quercetin in the presence of control siRNA, circHIAT1 siRNA or circHIAT1 siRNA plus miR-19a-3p inhibitor. qRT-PCR analysis showed that quercetin promoted circHIAT1 expression and circHIAT1 siRNA repressed its level. Quercetin reduced miR-19a-3p expression, and the transfection of circHIAT1 siRNA increased miR-19a-3p level; and miR-19a-3p inhibitor could also reduced miR-19a-3p level upon circHIAT1 silencing (Fig 5A). As expected, quercetin repressed the expression of N-cadherin and promoted E-cadherin level, which could be impaired by circHIAT1 siRNA; the co-transfection of miR-19a-3p inhibitor abrogated the effect of circHIAT1 siRNA (Fig 5B). Silencing circHIAT1 also abolished the inhibitory effect of quercetin on cell proliferation, while the effect of circHIAT1 knockdown was attenuated by miR-19a-3p inhibition (Fig 5C). In agreement with the change of EMT markers, silencing circHIAT1 rescued cell migration and invasion upon quercetin treatment, while the effect of circHIAT1 knockdown was abolished by miR-19a-3p inhibition (Fig 5D and 5E). Thus, the anti-cancer effect of quercetin depends on circHIAT1/miR-19a-3p axis.

### Quercetin re-sensitizes BC cells to CDK4/6 inhibitor Palbociclib by targeting circHIAT1

**Fig 6:**
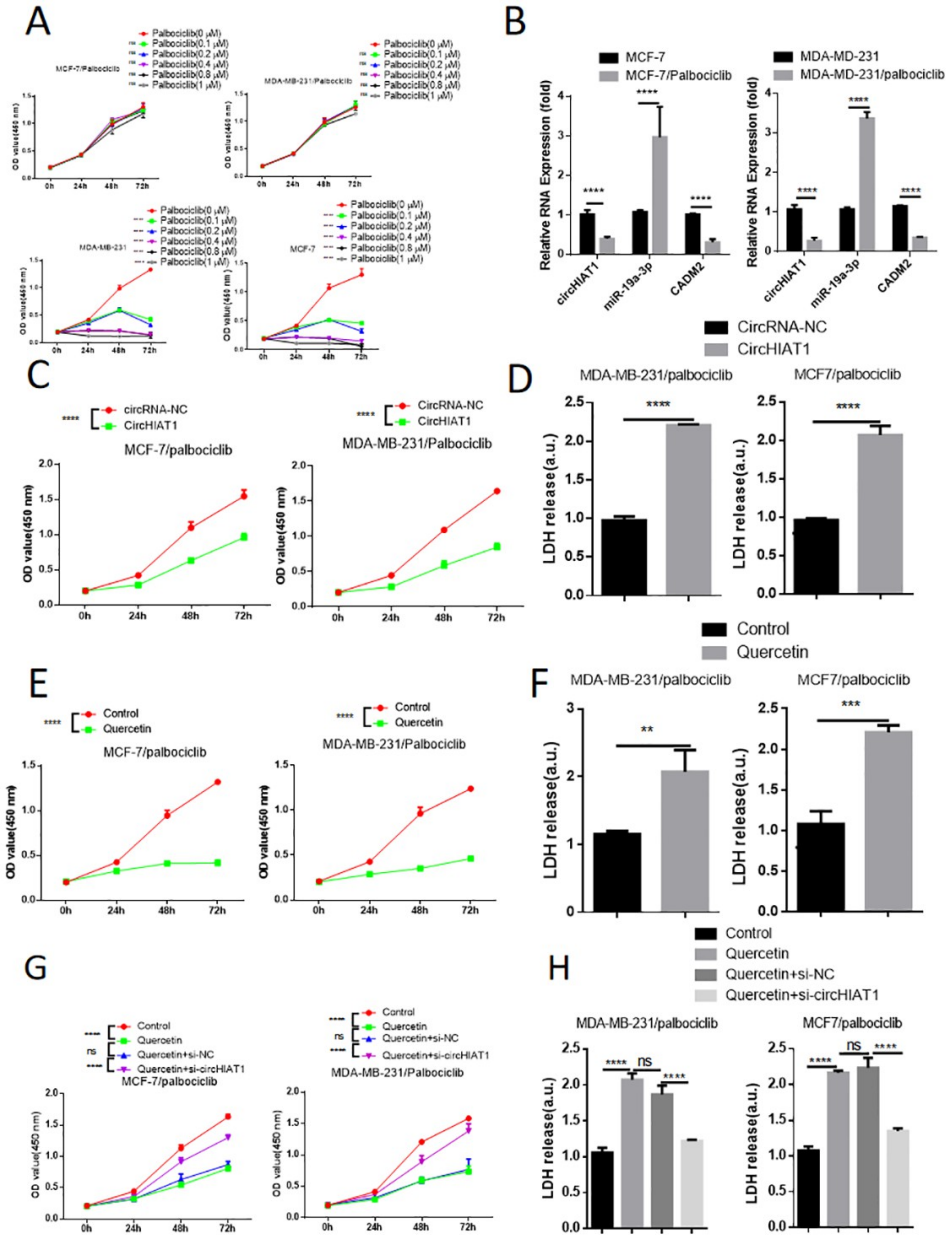
Quercetin re-sensitizes BC cells to CDK4/6 inhibitor Palbociclib by targeting circHIA. (A). To investigate the effect of quercetin on Palbociclib drug resistance, we continuously exposed BC cells to increasing doses of Palbociclib. CCK-8 assay showed that drug-resistant MCF7 and MDA-MB-231 cells were able to proliferate in the presence of different doses of Palbociclib. (B). qRT-PCR was performed to analyze circHIAT1, miR-19a-3p and CADM2 expression in parental and drug-resistant cells; (C). Drug-resistant BC cells were transfected with empty vector or circHIAT1 expression vector. CCK-8 assay was performed in the presence of 0.8 μM Palbociclib; (D). LDH cytotoxicity assay was performed in above (C) conditions; (E). To further study whether quercetin impacts on Palbociclib resistance, drug-resistant BC cells were treated with 10 μM quercetin for 24 hours before Palbociclib treatment. Cell proliferation ability of BC cells was assessed under 0.8 μM Palbociclib; (F). LDH cytotoxicity assay was performed in above (E) conditions; (G). CCK-8 proliferation assay was performed in drug-resistant BC cells treated with 10 μM quercetin and/or with circHIAT1 silencing. Palbociclib was applied at 0.8 μM in all conditions; (H). LDH cytotoxicity assay was performed in above (G) conditions. *p<0.05; **p<0.01; ***p<0.001; ****p<0.0001.

To investigate the effect of quercetin on Palbociclib drug resistance, we continuously exposed BC cells to increasing doses of Palbociclib. In contrast to the parental cell lines, the resistant MCF7 and MDA-MB-231 cells were able to proliferate in the presence of different doses of Palbociclib (Fig 6A). In the drug-resistant cells, we also found that circHIAT1 and CADM2 became down-regulated while miR-19a-3p was up-regulated (Fig 6B). We therefore investigated whether circHIAT1 dysregulation also contribute to Palbociclib resistance. Drug-resistant cells were transfected with empty vector or circHIAT1 expression vector. In the presence of 0.8 μM Palbociclib, drug-resistant cells continued to proliferate while circHIAT1 over-expression repressed the proliferation (Fig 6C). In addition, LDH assay revealed that circHIAT1 over-expression re-sensitized the resistant BC cells to Palbociclib induced cytotoxicity (Fig 6D). Therefore, circHIAT1 down-regulation contributes to Palbociclib drug resistance.

To further study whether quercetin impacts on Palbociclib resistance, drug-resistant cells were treated with 10 μM quercetin for 24 hours before Palbociclib treatment. Co-treatment with quercetin abrogated the cell proliferation ability of BC cells under 0.8 μM Palbociclib, and also induced cell cytotoxicity (Fig 6E and 6F). Since quercetin treatment increased circHIAT1 level in BC cells (Fig 4F), we silenced circHIAT1 to examine whether circHIAT1 up-regulation contributes to the effect of quercetin. circHIAT1 knockdown by siRNA suppressed the effect of quercetin on sensitizing BC cells to Palbociclib treatment (Fig 6G and 6H). Together, these data suggest that quercetin is able to re-sensitize drug-resistant BC cells to CDK4/6 inhibitor Palbociclib by targeting circHIAT1.

### Quercetin promotes Palbociclib sensitivity of drug-resistant BC cells in mouse xenograft model

**Fig 7:**
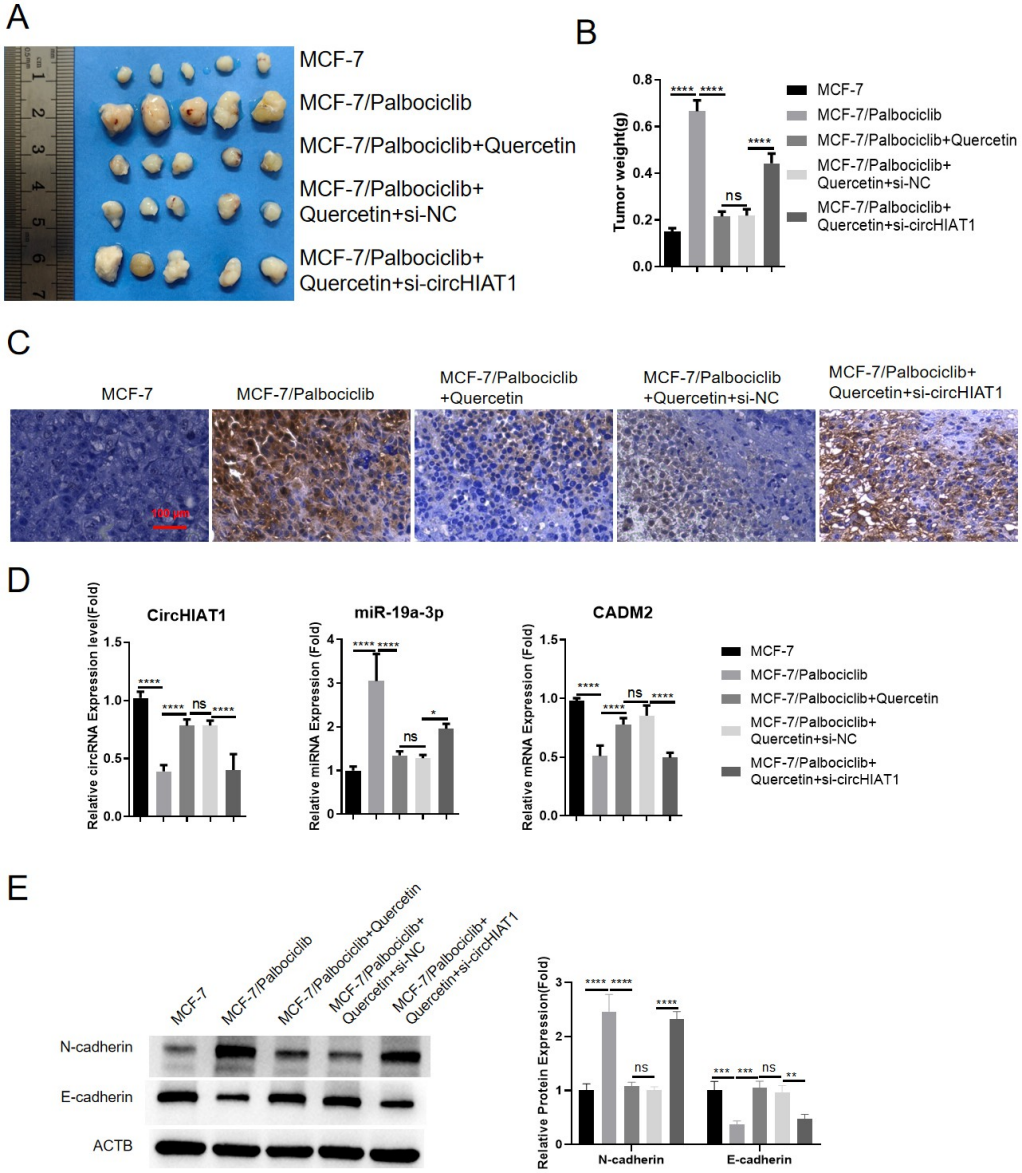
Quercetin boosts the anti-cancer effect of Palbociclib on BC cells in mouse xenograft model. To evaluate the in vivo effect of quercetin on drug-resistant BC cells, nude mice were injected with drug-sensitive or drug-resistant MCF-7 cells. The drug-resistant group was also administered with quercetin, or quercetin and circHIAT1 siRNA. All the mice were treated with Palbociclib to assess the drug sensitivity. (A). Images of xenograft tumors in each group; (B). Summary of tumor weight; (C). IHC staining of Ki-67 cell proliferation marker in tumor sections; (D). qRT-PCR was performed to analyze circHIAT1, miR-19a-3p and CADM2 expression in the tumor samples of each group. (E). Western blot analysis of N-cadherin and E-cadherin protein levels in the tumor samples of each group. *p<0.05; **p<0.01; ***p<0.001; ****p<0.0001.

In order to evaluate the *in vivo* effect of quercetin treatment on drug-resistant BC cells, nude mice were injected with drug-sensitive or drug-resistant MCF-7 cells. The drug-resistant group was also administered with quercetin, or quercetin and circHIAT1 siRNA. All the mice were treated with Palbociclib to assess the drug sensitivity. As expected, drug-resistant MCF-7 cell injection led to large tumor size and heavier tumor weight when compared to the mice injected with drug-sensitive cells; quercetin administration could retard tumor growth of drug-resistant MCF-7 cells, and this effect was attenuated by circHIAT1 siRNA (Fig 7A and 7B). The results of IHC staining in the tumor sections showed that the cells expressing the proliferation marker Ki67 were increased in drug resistant-tumor section, and the number of Ki67-positive cells was decreased after quercetin treatment, but the knockdown of circHIAT1 could reverse the effect of quercetin (Fig 7C). Further, qRT-PCR analysis revealed that circHIAT1 and CADM2 were down-regulated in drug-resistant cells, and quercetin treatment increased their expression. These effects were inhibited by circHIAT1 knockdown, while miR-19a-3p showed an opposite expression pattern (Fig 7D). Furthermore, the analysis of EMT markers demonstrated that, the elevated expression of mesenchymal marker N-cadherin in drug-resistant tumor was suppressed by quercetin treatment, and the effect of quercetin was partially abrogated upon circHIAT1 silencing. The expression pattern of E-cadherin exhibited an opposite trend to that of N-cadherin (Fig 7E). Thus, quercetin also suppresses the mesenchymal state of BC cells *in vivo* through modulating circHIAT1. Collectively, these results imply that quercetin boosts the anti-cancer effect of Palbociclib on drug-resistant BC cells by targeting circHIAT1.

## Discussion

As a major cause of cancer-related death and a common type of cancer in women, BC appears in a wide age range from 20–60 years old [26]. The prevalence of BC has increased noticeably over the past few years [27]. Although the advanced diagnosis and treatment solutions improved the overall survival of BC patients [28, 29], a large portion of patients suffers from dismal prognosis due to distance metastasis, drug resistance and cancer recurrence [30, 31]. In this study, we demonstrated the roles of circHIAT1/miR-19a-3p/CADM2 axis in modulating the EMT and drug resistance of Palbociclib in BC cells, and showed that quercetin can act as a potential bioactive compound to reverse Palbociclib resistance in BC treatment.

There is a strong relationship between therapeutic resistance and EMT induction in cancer [32]. CDK4/6 inhibitors have been developed as therapeutic agents for treating a wide range of malignancies [33]. Clinical evidence showed that about only 10% of the patients show primary resistance to CDK4/6 inhibitors, and the number of patients with failed treatment increase with prolonged drug administration [34]. Palbociclib has been implemented as a first-line therapy along with other endocrine drugs for BC treatment [35]. CDK4/6 inhibitor resistance is related to EMT through activating the PI3K/AKT/mTOR and TGF-β-Smad signaling pathways [11]. We showed that circHIAT1 and CADM2 becomes down-regulated while miR-19a-3p is over-expressed in Palbociclib resistant BC cells. circHIAT1 over-expression could impair EMT of BC cells and re-sensitizes drug-resistant BC cells to Palbociclib treatment *in vitro* and *in vivo*. We also demonstrated the molecular interplay of circHIAT1, miR-19a-3p and CADM2 in BC cells. Theretofore, circHIAT1/miR-19a-3p/CADM2 may play a critical role in dictating EMT process and Palbociclib drug resistance in BC cells.

Cell Adhesion Molecule 2 (CADM2) is engaged in homo- and heterophilic interactions with the other nectin-like family members. CADM2 over-expression could suppressed the EMT and cell proliferation of esophageal squamous cell carcinoma cells by repressing AKT signaling pathway [36]. CADM2 dysregulation has been also implicated in the regulation of EMT process of other types of cancers [37, 38]. In this study, we reported that down-regulation of CADM2 is associated with entranced EMT process in Palbociclib-resistant BC cells. We also provide evidence that circHIAT1/miR-19a-3p axis regulates CADM2 in BC cells. The mechanisms by which CADM2 regulates Palbociclib resistance in BC cells need to be further clarified in future work.

Quercetin has been reported to induced apoptosis and necrosis in BC cells [20, 21, 39]. However, in this study, we demonstrated that at sub-toxic dose, quercetin treatment could impair EMT and re-sensitize drug-resistant BC cells to Palbociclib treatment. This effect seems to be dependent on its effect on inducing circHIAT1 up-regulation, which then impinges on miR-19a-3p and CADM2 expression. Many studies have reported the role of miR-19a-3p role in BC. For instance, Xiang et al reported that miR-19a-3p up-regulation promotes the migration and EMT in BC cells, and dampens the sensitivity to Letrozole [23]. There is also evidence that miR-19 enhances cell invasion, proliferation, migration, and EMT in osteosarcoma and lung cancer cells [40, 41]. Jiang et al. highlighted the role of miR-19a-3p in promoting chemoresistance and metastasis in hepatocellular carcinoma by targeting PTEN/AKT pathway [42]. Therefore, our data are consistent with the notion that miR-19a-3p serves as an oncogenic factor to promote the progression of BC cells by augmenting EMT and drug resistance. However, how quercetin induces circHIAT1 up-regulation in BC cells requires future investigation.

There are very few reports on the relationship between circHTAT1 and miR-19a-3p. According to Hu et al., miR-19a-3p was extensively expressed in cervical and circHIAT1 was a sponge factor of miR-19a-3p to suppress its activity [43]. The tumor-suppressive function of circHIAT1 was also reported in hepatocellular carcinoma [44]. Yang et al. showed that miR-19a-3p was up-regulated significantly in renal carcinoma and CADM2 was a direct target of miR-19a-3p [24]. The knockdown of miR-19a-3p suppressed the invasion, proliferation, and EMT of renal carcinoma cells. In current study, we found that quercetin suppressed the migration, invasion, and EMT in BC cells, while circHIAT1 knock-down impaired the effect of quercetin. As far as we know, this is the first report regarding the role of quercetin on the EMT and CDK4/6 inhibitor drug resistance in BC. Although palbociclib was known to inhibit the EMT and metastasis in BC [9], the prevalent drug resistance undermines its treatment outcome. Our findings suggest that the joint application of quercetin and palbociclib might have synergistic effect on suppressing the EMT and metastasis in BC.

## Conclusions

Taken together, our study identified quercetin as a potential anti-cancer compound to reverse Palbociclib resistance and impair EMT in BC cells by targeting circHIAT1/miR-19a-3p/CADM2 axis. These data not only provide novel insights into the regulation of EMT and invasive features of BC cells, but also hint on the potential of joint application of quercetin and palbociclib to overcome drug resistance. Future work needs to clarify the mechanisms by which quercetin impinges on circHIAT1/miR-19a-3p/CADM2 axis.

## Supporting information

S1 Raw data (DOCX)
