# Supplementary material for: Quercetin inhibits the epithelial-mesenchymal transition and reverses CDK4/6 inhibitor resistance in breast cancer by regulating circHIAT1/miR-19a-3p/CADM2 axis
Source: PLoS One. 2024 Jul 11;19(7):e0305612. doi: 10.1371/journal.pone.0305612 (PMC11239024; doi:10.1371/journal.pone.0305612)

Figure 2


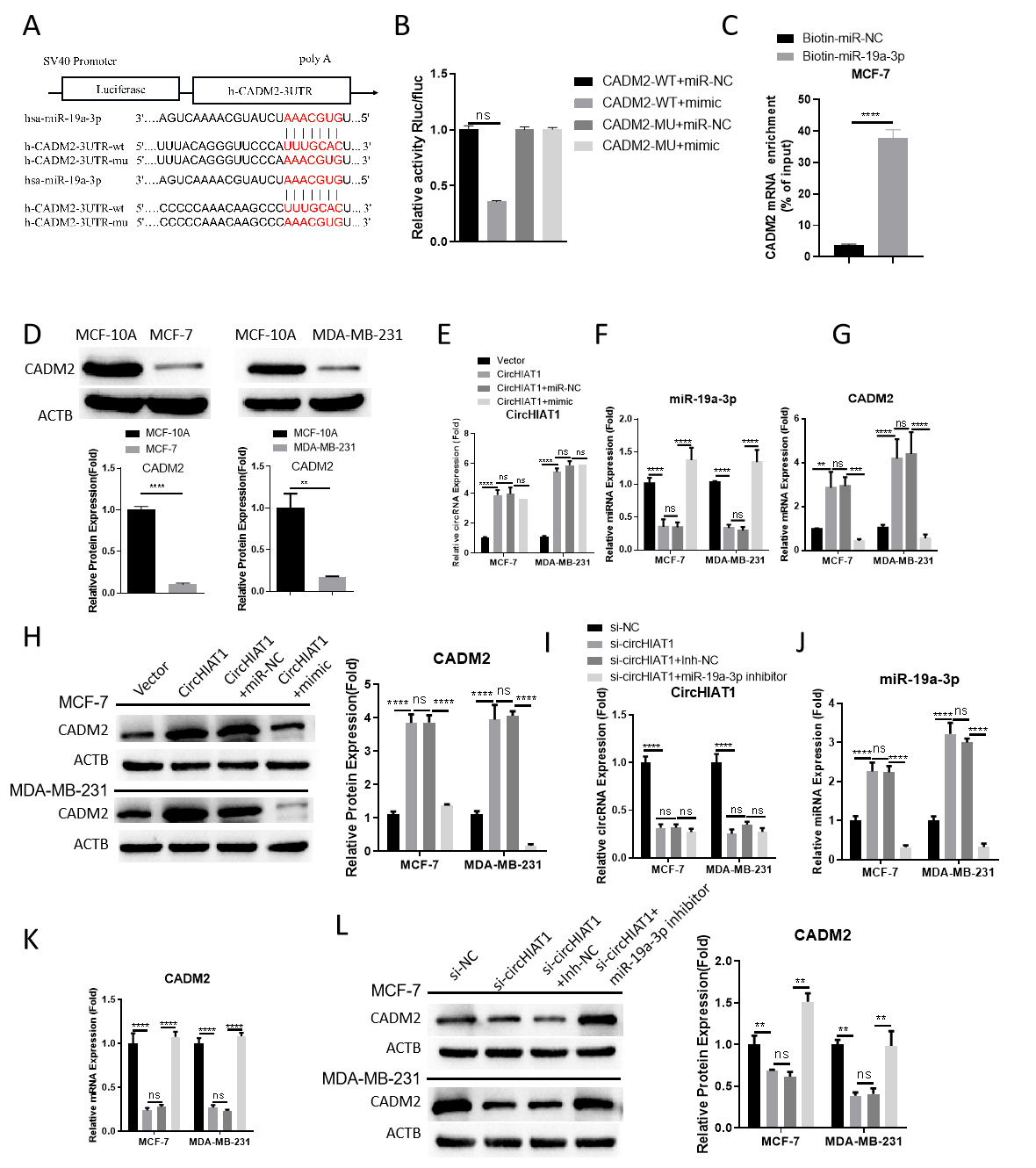


Figure 2D-MCF-7

CADM2


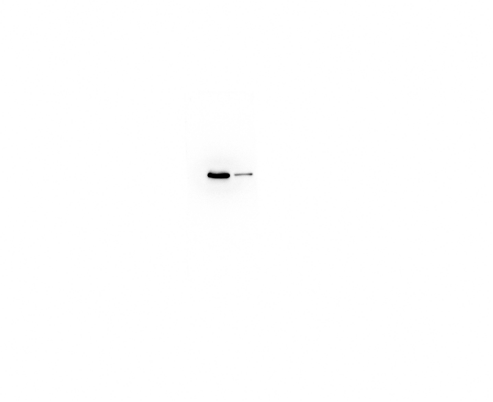

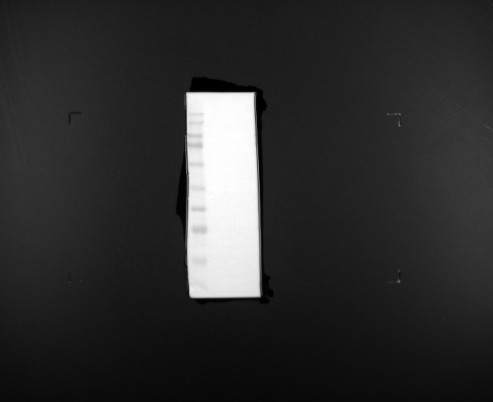


ACTB







Figure 2D-MDA-MB-231

CADM2


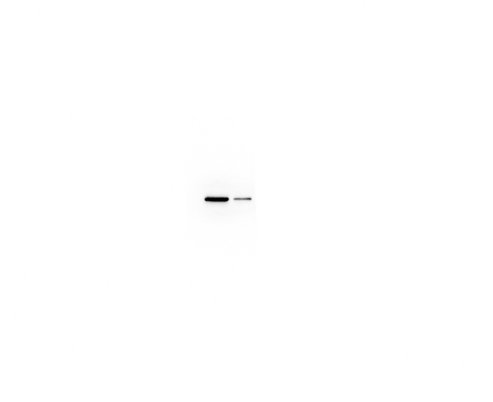

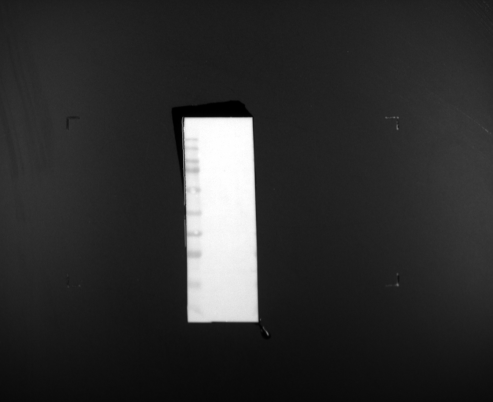


ACTB




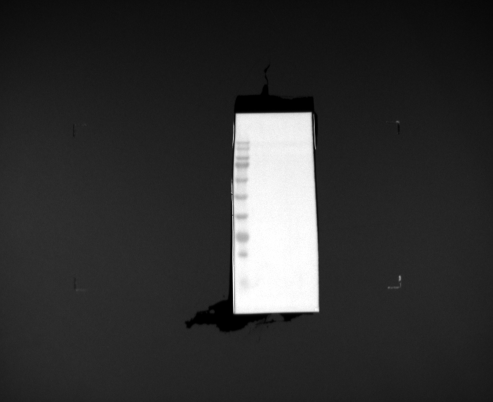


Figure 2H-MCF-7

CADM2







ACTB







Figure 2H- MDA-MB-231

CADM2







ACTB


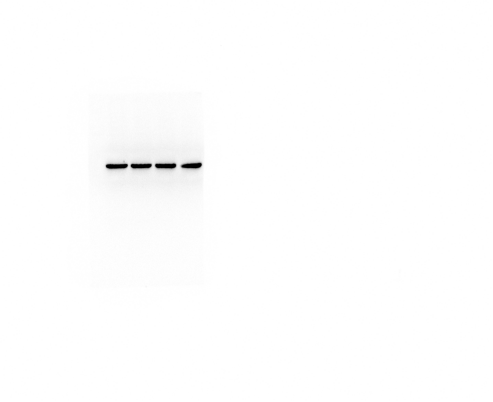

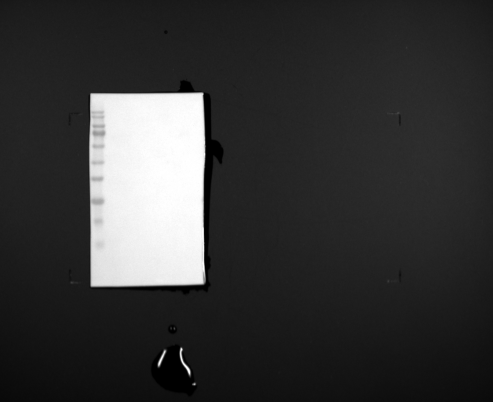


Figure 2L-MCF-7

CADM2


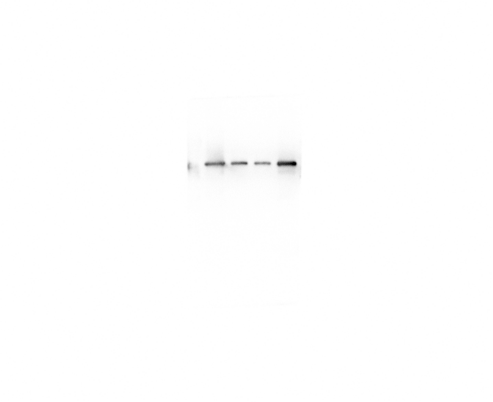

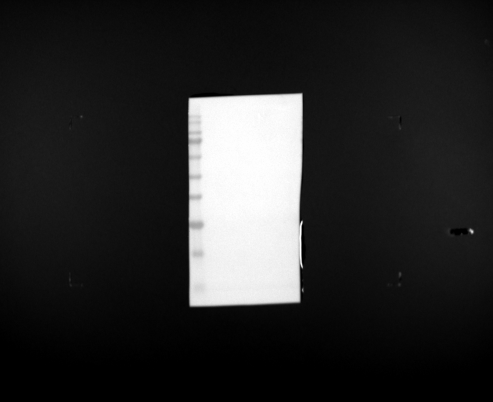


ACTB


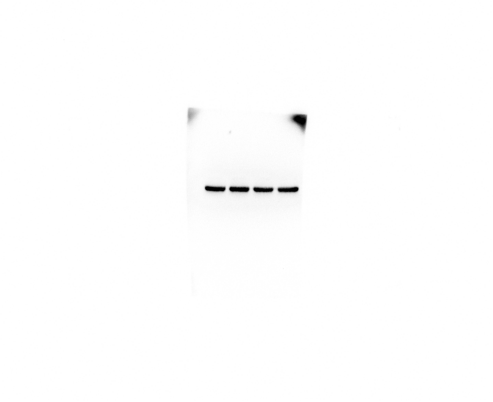

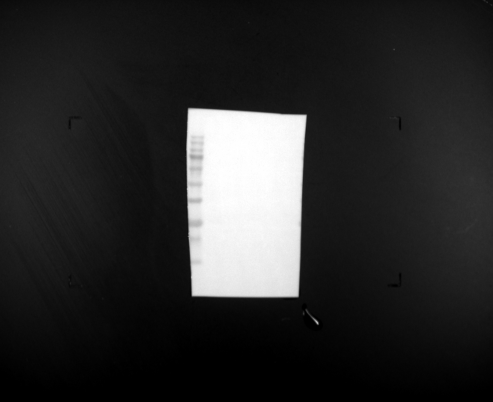


Figure 2L- MDA-MB-231

CADM2


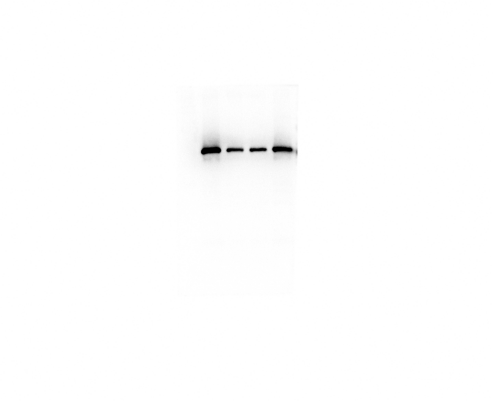

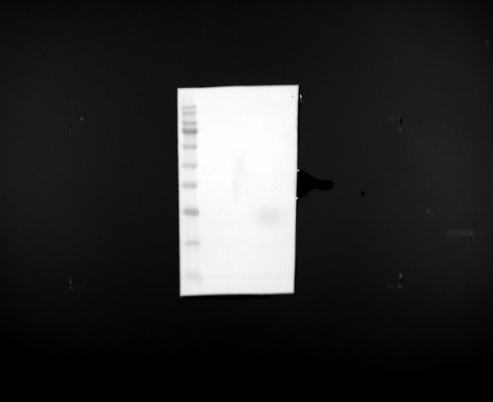


ACTB


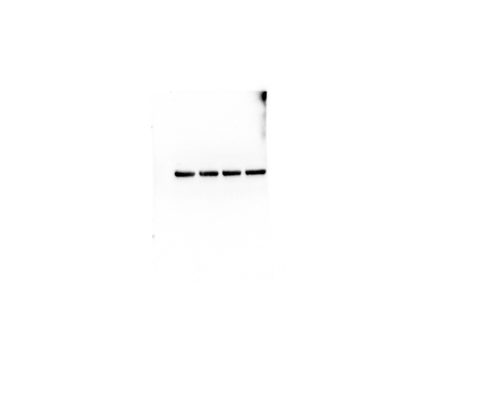

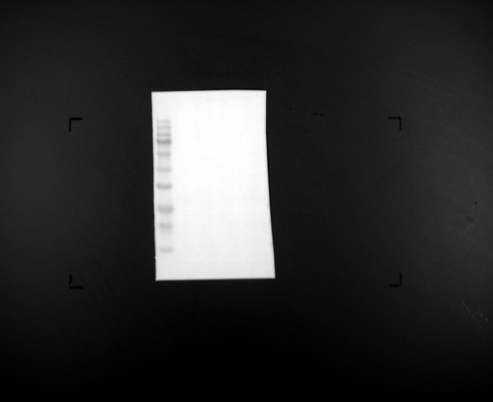


Figure 3


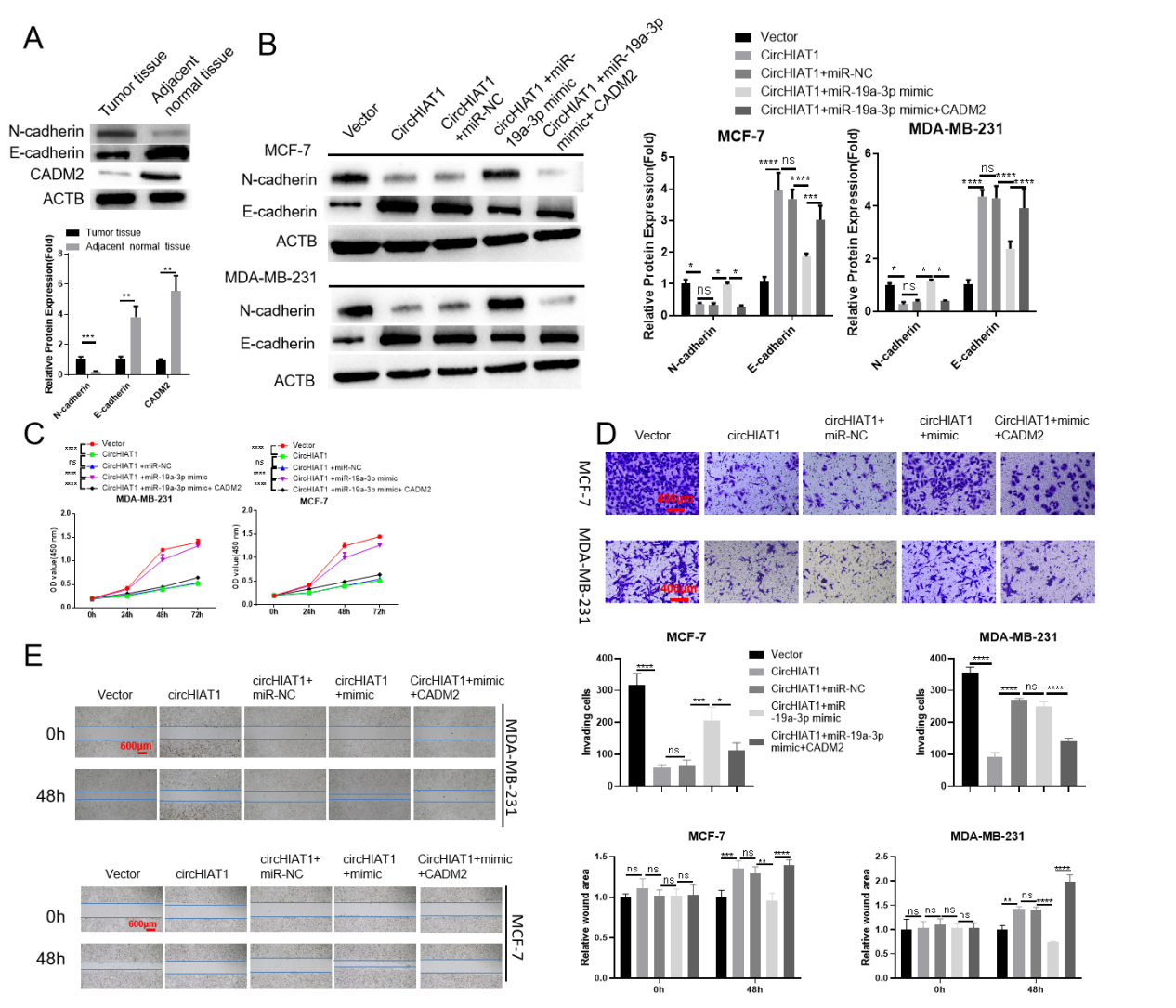


Figure 3A

N-cadherin


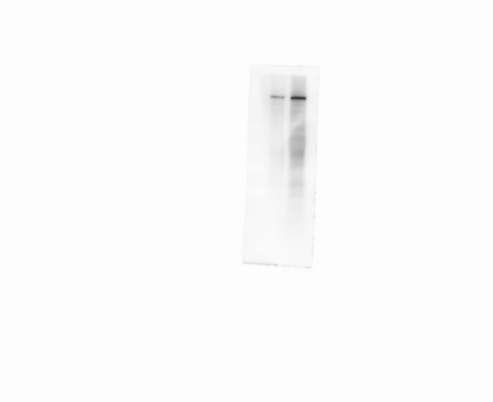




E-cadherin


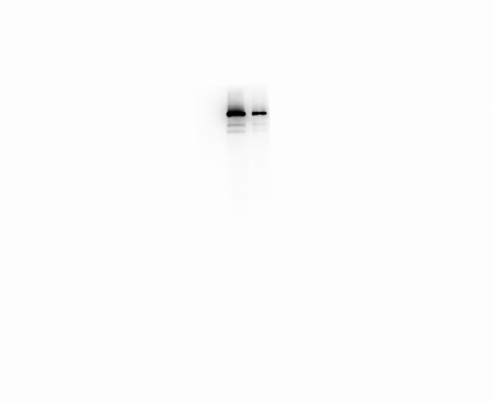




CADM2


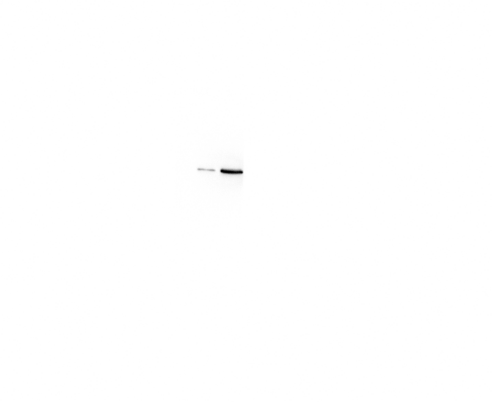

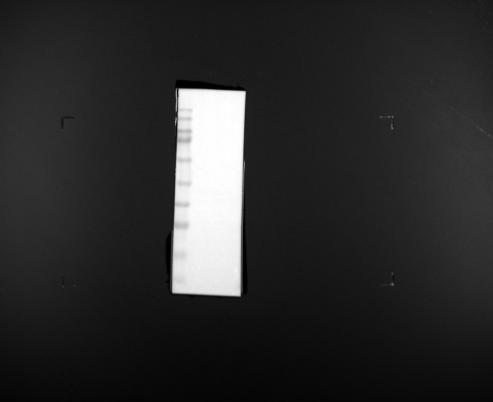


ACTB







Figure 3B-MCF-7

N-cadherin


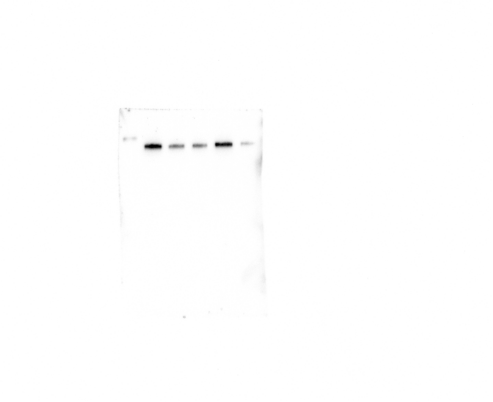

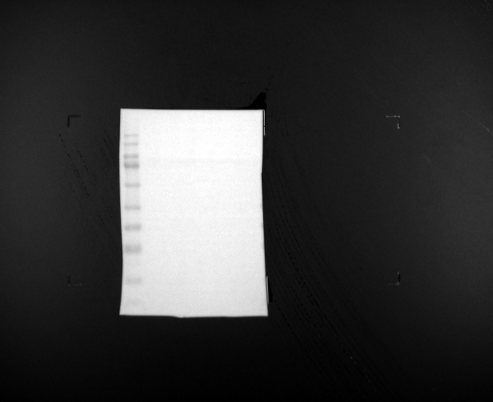


E-cadherin







ACTB







Figure 3B-MDA-MB-231

N-cadherin


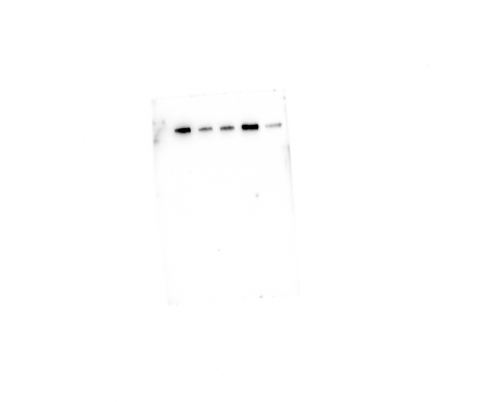

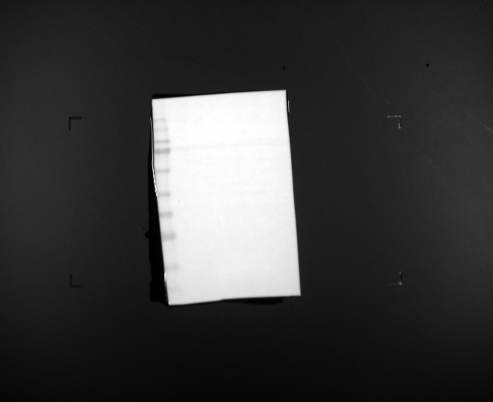


E-cadherin







ACTB







Figure 4


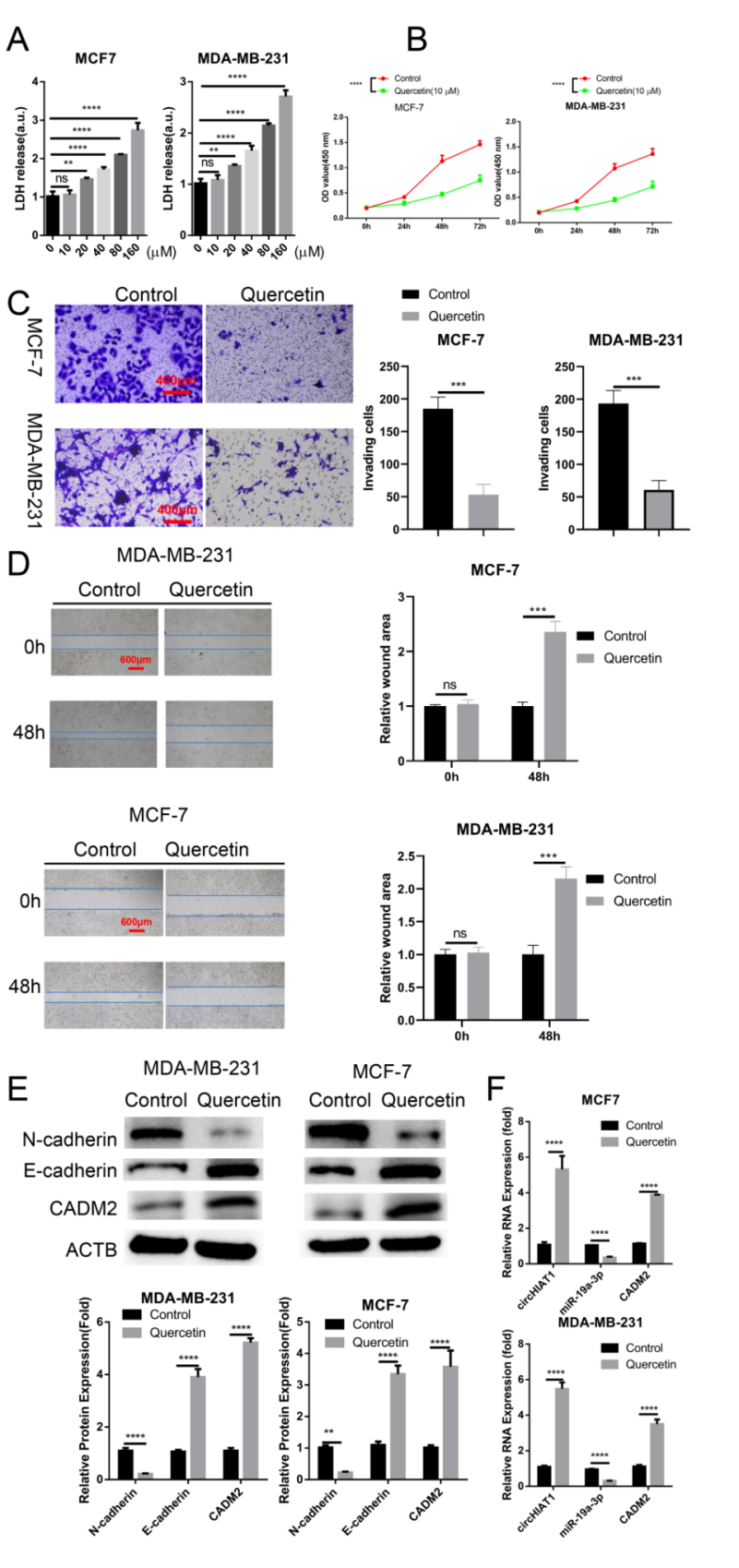


Figure 4E-MCF-7

N-cadherin







E-cadherin


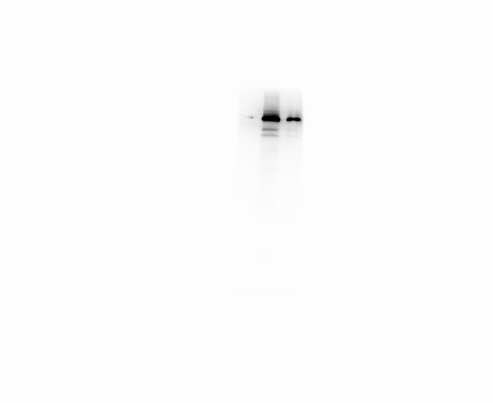




CADM2 of MCF-7 and MDA-MB-231


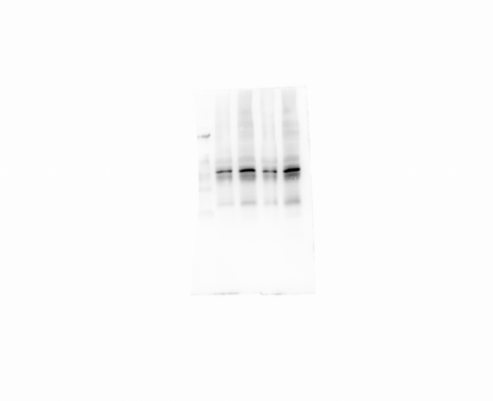




ACTB







Figure 4E-MDA-MB-231

N-cadherin







E-cadherin


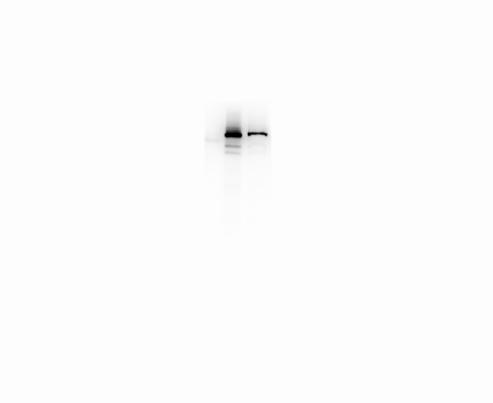




ACTB


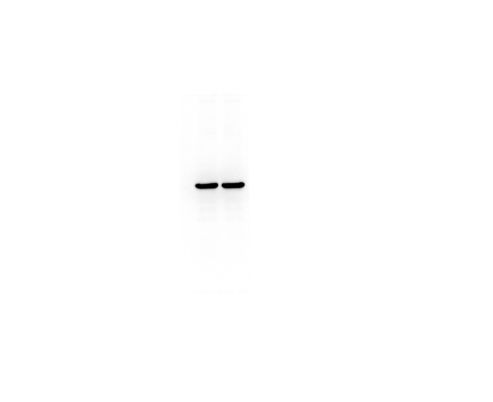

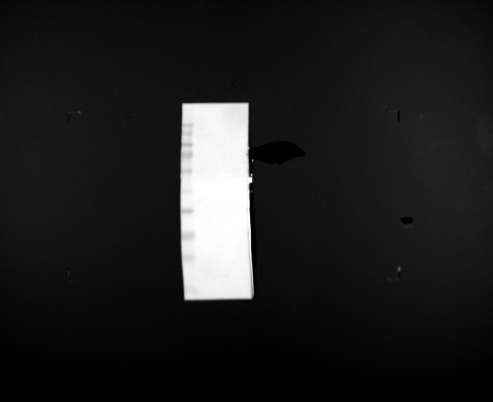


Figure 5


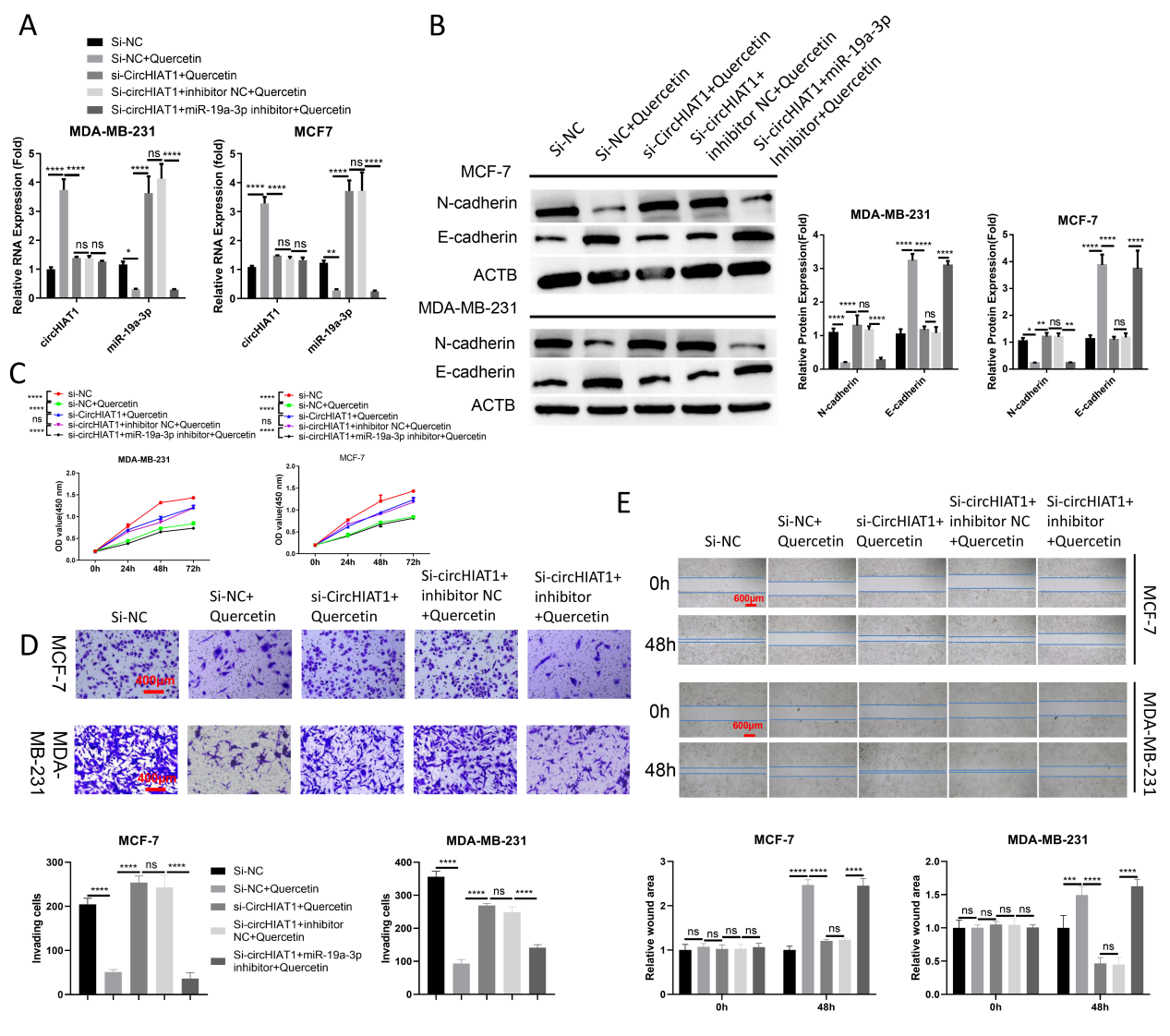


Figure 5B-MCF-7

N-cadherin







E-cadherin







ACTB







Figure 5B-MDA-MB-231

N-cadherin


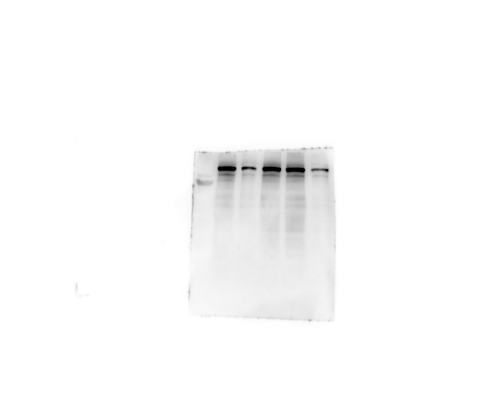

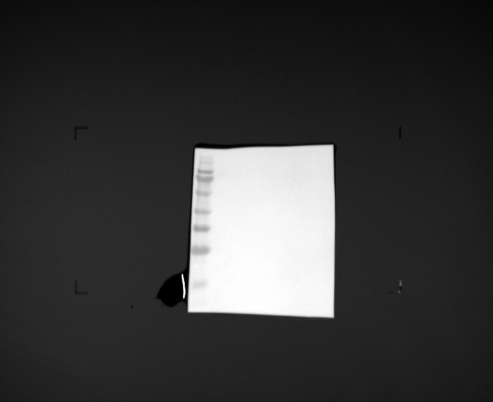


E-cadherin







ACTB







Figure 7


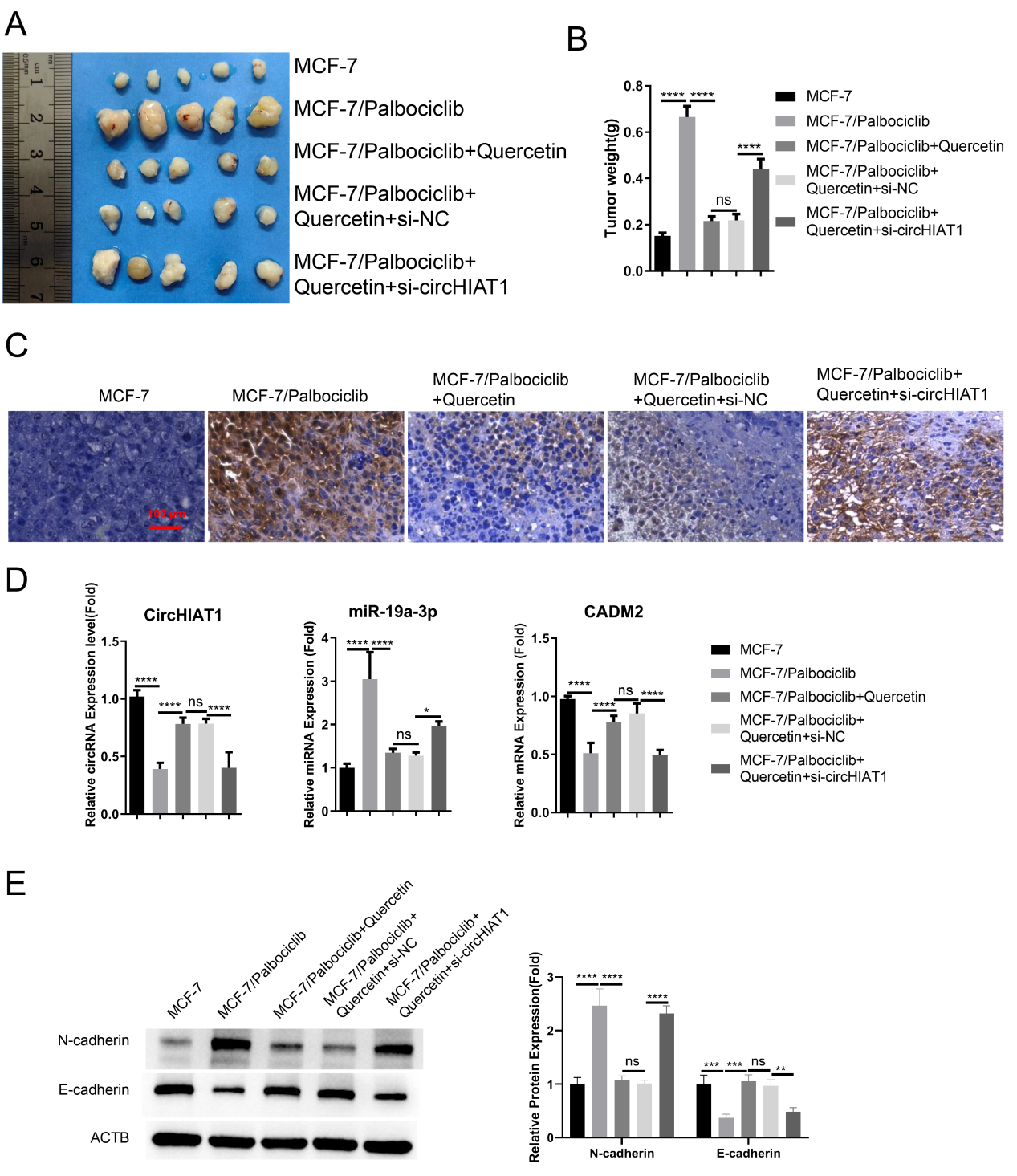


Figure 7E

1. Cadherin


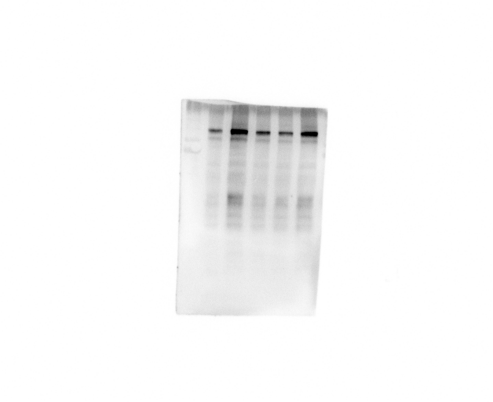

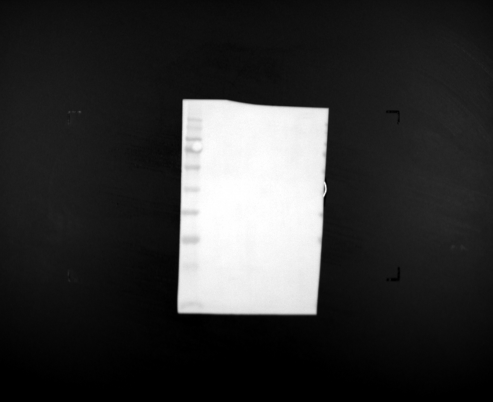


1. Cadherin


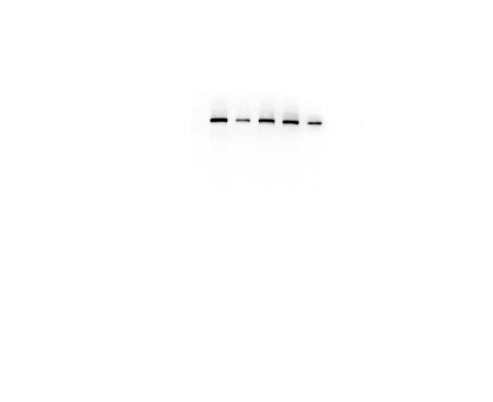

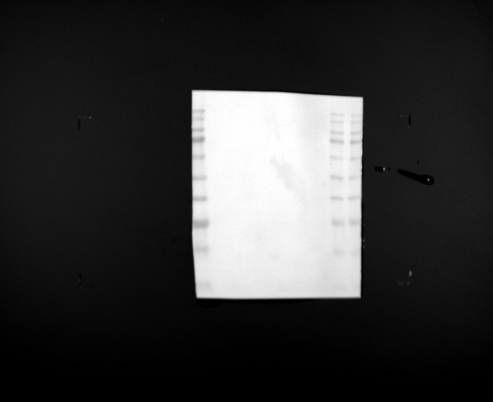


ACTB


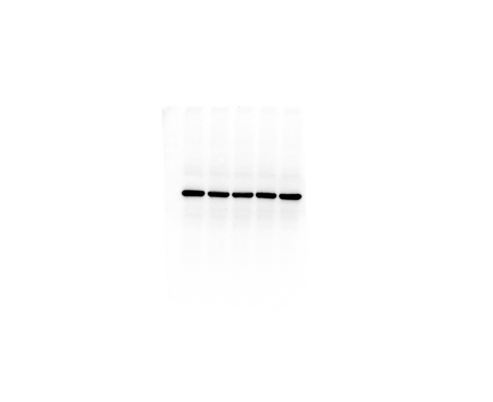

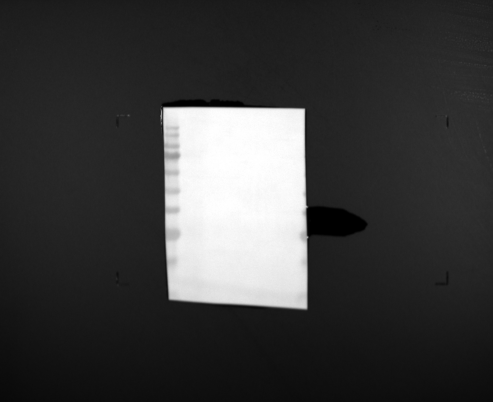

Supplement: S1 Raw data — (DOCX) [file pone.0305612.s001.docx]
